# Contrasting Evolutionary Patterns Between Sexual and Asexual Lineages in a Genomic Region Linked to Reproductive Mode Variation in the pea aphid

**DOI:** 10.1093/gbe/evad168

**Published:** 2023-09-17

**Authors:** Maud Rimbault, Fabrice Legeai, Jean Peccoud, Lucie Mieuzet, Elsa Call, Pierre Nouhaud, Hélène Defendini, Frédérique Mahéo, William Marande, Nicolas Théron, Denis Tagu, Gaël Le Trionnaire, Jean-Christophe Simon, Julie Jaquiéry

**Affiliations:** INRAE, UMR 1349, Institute of Genetics, Environment and Plant Protection, Le Rheu, France; INRAE, UMR 1349, Institute of Genetics, Environment and Plant Protection, Le Rheu, France; University of Rennes, Inria, CNRS, IRISA, Rennes, France; Laboratoire Ecologie et Biologie des Interactions, Equipe Ecologie Evolution Symbiose, Unité Mixte de Recherche 7267 Centre National de la Recherche Scientifique, Université de Poitiers, Poitiers CEDEX 9, France; INRAE, UMR 1349, Institute of Genetics, Environment and Plant Protection, Le Rheu, France; INRAE, UMR 1349, Institute of Genetics, Environment and Plant Protection, Le Rheu, France; INRAE, UMR 1349, Institute of Genetics, Environment and Plant Protection, Le Rheu, France; CBGP, INRAE, CIRAD, IRD, Montpellier SupAgro, Univ Montpellier, Montpellier, France; INRAE, UMR 1349, Institute of Genetics, Environment and Plant Protection, Le Rheu, France; INRAE, UMR 1349, Institute of Genetics, Environment and Plant Protection, Le Rheu, France; French Plant Genomic Resource Center, INRAE-CNRGV, Castanet Tolosan, France; French Plant Genomic Resource Center, INRAE-CNRGV, Castanet Tolosan, France; INRAE, UMR 1349, Institute of Genetics, Environment and Plant Protection, Le Rheu, France; INRAE, UMR 1349, Institute of Genetics, Environment and Plant Protection, Le Rheu, France; INRAE, UMR 1349, Institute of Genetics, Environment and Plant Protection, Le Rheu, France; INRAE, UMR 1349, Institute of Genetics, Environment and Plant Protection, Le Rheu, France

**Keywords:** life-cycle, reproductive polymorphism, sexual reproduction, asexuality, genome scan, cyclical parthenogenesis

## Abstract

Although asexual lineages evolved from sexual lineages in many different taxa, the genetics of sex loss remains poorly understood. We addressed this issue in the pea aphid *Acyrthosiphon pisum*, whose natural populations encompass lineages performing cyclical parthenogenesis (CP) and producing one sexual generation per year, as well as obligate parthenogenetic (OP) lineages that can no longer produce sexual females but can still produce males. An SNP-based, whole-genome scan of CP and OP populations sequenced in pools (103 individuals from 6 populations) revealed that an X-linked region is associated with the variation in reproductive mode. This 840-kb region is highly divergent between CP and OP populations (*F_ST_* = 34.9%), with >2,000 SNPs or short Indels showing a high degree of association with the phenotypic trait. In OP populations specifically, this region also shows reduced diversity and Tajima’s *D*, consistent with the OP phenotype being a derived trait in aphids. Interestingly, the low genetic differentiation between CP and OP populations at the rest of the genome (*F_ST_* = 2.5%) suggests gene flow between them. Males from OP lineages thus likely transmit their *op* allele to new genomic backgrounds. These genetic exchanges, combined with the selection of the OP and CP reproductive modes under different climates, probably contribute to the long-term persistence of the *cp* and *op* alleles.

**Significance**

Asexual taxa occur in all major clades of eukaryotes and derive from sexual species. Yet, the genetic basis of these transitions is poorly understood because crosses cannot generally be performed to genetically map the ability to propagate asexually. As a result, a gene presumably responsible for sex loss has been identified in only one animal species—the Cape honeybee. Here, using pooled genome sequencing, we identified a 840-kb region (carrying 32 genes) that is associated with the transition to permanent asexuality in the pea aphid. We also revealed that sexual and asexual alleles likely diverged several hundred thousand years ago and that asexual lineages probably persist through contagious asexuality, whereby the few males they produce transmit asexual genes to sexual lineages. These results provide new insights into the mechanisms of coexistence of sexual and asexual lineages.

## Introduction

The prevalence of sexual reproduction in eukaryotes (Bell 1982) has long been considered as an evolutionary paradox, because sexual organisms transmit their genetic information twice less efficiently as asexual organisms do (Maynard Smith 1971). There is now a consensus that sex is favored over asexuality in the long term because it purges deleterious mutations that otherwise accumulate in asexual genomes, combines favorable mutations into genomes faster and generates genotypic diversity fueling adaptation (Muller 1964; Barton and Charlesworth 1998). Indeed, only few ancient asexual lineages exist (e.g., Mark Welch and Meselson 2000; Martens et al. 2003), indicating the inability of asexual lineages to persist over long evolutionary time due to long-term costs. However, how sex is maintained in the short term when sexual and asexual lineages coexist is still under debate (Hartfield and Keightley 2012). The loss of sexual reproduction is observed in many animal taxa such as squamates, fishes, insects, crustaceans, nematodes, and mollusks (Vrijenhoek et al. 1989; Schon et al. 2009). These frequent transitions from sexual to asexual reproduction reflect well the theoretical demographic advantage of asexual lineages over their sexual counterparts, which may allow them to persist over ecological times.

Sex may be lost by different ways (including interspecific hybridization, microorganism infection, spontaneous mutation, or spread of contagious asexuality elements) and at various frequency. The mechanisms of these losses affect the genetic features of the derived asexual lineages (Simon et al. 2003; van der Kooi and Schwander 2014). However, little is known about the genes underlying the shifts to asexuality. Indeed, one cannot use standard crossing techniques to genetically map the ability to propagate asexually (Neiman et al. 2014). Remarkably, certain species present lineages that have only partially lost sexual reproduction, allowing the identification of the genetic basis of sex loss using recombination-based approaches. Such crosses have revealed that the genetic mechanism responsible for the transitions from cyclical to obligate parthenogenesis in aphids (Dedryver et al. 2013; Jaquiéry et al. 2014), rotifers (Stelzer et al. 2010), and cladocerans (Lynch et al. 2008; Tucker et al. 2013; Xu et al. 2015), and from arrhenotoky to thelytoky in hymenopterans (Lattorff et al. 2005, 2007; Sandrock and Vorburger 2011; Aumer et al. 2017, 2019) involves only one or a few loci. However, in most cases, the precise location, the nature, and function of the genetic determinants of these shifts to obligate asexuality remain largely unknown.

The Cape honeybee is the animal species in which the gene responsible for sex loss is best characterized. Queens (and workers under certain conditions) produce haploid males via arrhenotokous parthenogenesis. Nevertheless, some workers in the Cape honeybee have the ability to produce diploid eggs through thelytokous parthenogenesis. The extensive research into the genetic basis of this trait has yielded conflicting outcomes regarding the number of loci, the identification of the candidate gene, and the dominance/recessivity of the trait (Lattorff et al. 2005, 2007; Chapman et al. 2015; Wallberg et al. 2016; Aumer et al. 2017, 2019; Christmas et al. 2019, Yagound et al. 2020). The latest investigation points to a single gene (GB45239) that would be associated with thelytokous reproduction in workers (Yagound et al. 2020), the thelytokous allele being recessive. This gene encodes a protein that has structural similarity to SMC proteins, which typically play a role in chromosome assembly, segregation, and adhesion of sister chromatids. The functional characterization of the allelic variants at this candidate gene is crucial for establishing a causal link with thelytoky, given the controversy (Aumer et al. 2019; Christmas et al. 2019; Yagound et al. 2020).

Another well-studied system is *Daphnia pulex*, a crustacean reproducing by cyclical parthenogenesis, an alternation of many parthenogenetic generations and one sexual generation producing diapausing eggs, referred to as CP. In this species, sex-limited meiosis-suppressing genetic factors enable some lineages to produce diapausing eggs by parthenogenesis. These obligatory parthenogenetic lineages are called OP lineages. Genome sequencing of OP and CP lineages revealed that all *D. pulex* OP lineages share the same haplotypes in at least four genomic regions including almost two entire chromosomes and parts of two others (Lynch et al. 2008; Tucker et al. 2013; Xu et al. 2015), which have been acquired by hybridization with the close species *D. pullicaria*.

The identification of candidate loci for sex loss can also shed light on the origins and evolutionary dynamics of asexual lineages and/or asexual alleles. In the Cape honeybee, the allele associated with thelytoky appears to have emerged in this species and corresponds to a derived state (Yagound et al. 2020). In *D. pulex*, the large size of genomic regions associated with OP complicates the identification of candidate genes. However, some OP lineages still produce males, which can transmit the factors enabling permanent parthenogenetic reproduction when they mate with females from a CP lineage. These events create new OP lineages by so-called “contagious asexuality”. Analyses of rates of SNP conversion between OP and CP haplotypes within lineages revealed that all OP lineages of *D. pulex* were extremely young (22 years on average, Tucker et al. 2013). In contrast, the origin of the OP alleles is much older. Based on the synonymous divergence between the different OP haplotypes, it was estimated to have occurred between 1,250 and 187,000 years ago, corresponding to the divergence of the OP haplotypes clade from the homologous sequences in the exclusively sexual species *D. pulicaria* (Tucker et al. 2013). These results illustrate that, under contagious asexuality, the asexuality-conferring allele can be markedly older than OP lineages themselves. Even though each OP lineage might be doomed to extinction, the ancient asexual allele can persist by spreading in new genomic backgrounds through males.

Aphids are another appropriate model for studying the genetic basis of the loss of sex. The ancestral mode of reproduction in this group is CP, but nearly 45% of the 5,000 aphid species have partially or completely lost sexual reproduction (Moran 1992). Typically, CP lineages undergo several successive generations of parthenogenesis (by viviparous parthenogenetic females) in spring and summer. In autumn, photoperiod shortening triggers the production of oviparous sexual females and males (Le Trionnaire et al. 2008). The winter-diapausing eggs resulting from sexual reproduction are the only frost-resistant stage of the aphid developmental cycle (Simon et al. 2002). They give birth to viviparous parthenogenetic females in the next spring, which start a new cycle.

Interestingly, some lineages have lost the ability to produce sexual females in response to the photoperiodic cues, and thus reproduce yearlong by viviparous parthenogenesis (Simon et al. 2002, 2010; Frantz et al. 2006). These OP lineages are demographically advantaged over CP lineages in mild winter regions, mainly because they do not go through a long egg diapause. However, they cannot survive in regions with harsh winters because they are unable to produce cold-resistant eggs (Moran 1992). Thus, selection by climate results in a geographical distribution of reproductive phenotypes where OP lineages occupy regions with mild winters and CP lineages those with cold winters (Defendini et al. 2023), both co-occurring in areas with intermediate or fluctuating climates (Rispe and Pierre 1998; Simon et al. 2002, 2010). Interestingly, many OP lineages have retained the capacity to produce males in autumn, so that gene flow between OP and CP lineages may occur in the wild (Halkett et al. 2008; Dedryver et al. 2013; Jaquiéry et al. 2014). In addition, since OP-produced males are usually fertile (Dedryver et al. 2019; Defendini et al. 2023), they can be crossed with CP females to identify the genetic basis of reproductive mode variation.

In the pea aphid *Acyrthosiphon pisum*, such crosses have revealed that the OP phenotype was recessive (Jaquiéry et al. 2014). The combination of two complementary approaches—QTL mapping and low-resolution genome scan using microsatellite markers on populations submitted to divergent selection for reproductive mode—pinpointed a 10-cM genomic region located on the X chromosome associated with this trait (Jaquiéry et al. 2014). However, none of the ∼24,000 scaffolds constituting the ∼540-Mb pea aphid genome sequence was anchored to any of the four chromosomes (IAGC 2010) and most of the scaffolds longer than 150 kb contained assembly errors associating unlinked chromosomal regions (Jaquiéry et al. 2018). As a result, the genomic context of microsatellites linked to reproductive phenotypes could not be established. The recent release of an improved assembly of the pea aphid genome (Li et al. 2019), in which the four largest scaffolds correspond to the four chromosomes, provides an excellent opportunity to resolve this issue.

This study aims at finely characterizing the genomic region(s) associated with the variation of reproductive mode in the pea aphid and gaining functional and evolutionary insights into the genetic determinants of the loss of sex. To this end, we performed a high-resolution genome scan based on a pooled sequencing of 103 individuals from three OP and three CP populations. These individual samples had already been used in the previous low-resolution genome scan based on 439 microsatellite loci that identified one main candidate region associated with reproductive mode variation in *A. pisum* (Jaquiéry et al. 2014). The improved genome assembly combined with the millions of SNP markers scattered through the genome led to the identification of a major 840-kb genomic region showing strong genetic differentiation between OP and CP populations, and locating within the QTL locus previously identified by Jaquiéry et al. (2014). A thorough analysis of the variants present in this region was performed in an attempt to narrow down the list of candidate genes underlying the variation in reproductive mode in the pea aphid and to approximate the divergence time between the *op* and *cp* alleles.

## Results

### Genetic Structure of OP and CP Populations

A total of three OP populations, each consisting of 14 genetically distinct *A. pisum* lineages, and three CP populations, each composed of 20–21 lineages, were collected in alfalfa fields in Eastern Europe as described in Jaquiéry et al. (2014). These six populations were sequenced in pool, with two replicates per population, resulting in a total of 12 Pool-seq libraries. The number of 100 bp Illumina pair-end reads obtained ranged from 12 to 16 million per library, and from 24 to 28 million per population (table 1). The depth of sequencing, which varied from 15.1 to 20.4 depending on the libraries, enabled a total of 11 million SNPs to be identified. The PCA analysis of these SNPs separated the three OP populations from the three CP populations on the first axis, while the second axis mainly distinguished the OP populations (supplementary file 1, Supplementary Material online). As expected, sequencing replicates of the same population grouped together. Pairwise *F_ST_* analyses revealed no differentiation between pairs of populations of the same reproductive mode. However, there was a slight differentiation between reproductive modes, with an average *F_ST_* of 0.009 (see supplementary file 2, Supplementary Material online for more detailed information per population). When populations were grouped by reproductive mode (enabling a more precise estimate of allelic frequencies in each reproductive mode), the genome-wide *F_ST_* reached 0.025 between the two reproductive modes.

**Table 1:** Geographical Origins of the *Acyrthosiphon pisum* Populations Collected on *Medicago sativa*

Reproductive mode	Location	Pop ID	Latitude/Longitude	Number of lineages per pool	Library ID	Number of sequenced pairs	Number of mapped pairs	Properly paired	Properly paired w/o dups	Mean depth per library
CP	Saint-Prex—Switzerland	Sl	46°28' N 6°26' E	21	Sl02 Sl08	156,636,276 124,231,404	152,675,721 (97.5%) 120,516,514 (97.0)	144,341,466 (92.2%) 114,359,174 (92.1%)	140,910,102 (90.0%) 112,081,028 (90.2%)	20.4 16.2
	Ranspach—France	Vl	48°01'N 7°33' E	20	Vl03 Vl09	150,578,562 129,361,706	143,861,689 (95.5%) 124,600,198 (96.3%)	135,517,068 (90.0%) 117,371,582 (90.7%)	132,775,984 (88.2%) 115,159,748 (89.0%)	18.8 16.2
	Mirecourt—France	Mil	48°16'N 6°06' E	20	Mil01 Mil07	130,335,444 139,252,574	128,553,839 (98.6%) 134,680,780 (96.7%)	122,795,378 (94.21%) 127,537,064 (91.6%)	120,219,908 (92.2%) 124,638,190 (89.5%)	17.3 17.9
OP	Castelnaudary—France	Cast	43°19'N 1°57' E	14	Cast04 Cast10	144,133,842 121,695,798	138,947,572 (96.4%) 115,774,856 (95.1%)	131,604,542 (91.3%) 109,214,406 (89.7%)	129,063,056 (89.5%) 107,442,998 (88.3%)	18.8 15.6
	Gers—France	Gers	43°57'N 0°22' E	14	Gers05 Gers11	121,090,996 117,002,074	118,075,107 (97.5%) 113,374,313 (96.9%)	110,620,444 (91.4%) 106,802,538 (91.3%)	108,617,158 (89.7%) 104,951,584 (89.7%)	15.8 15.1
	Lusignan—France	Lus	46°24'N 0°04' E	14	Lus06 Lus12	122,039,296 133,230,226	117,497,862 (96.3%) 129,092,091 (96. 9%)	110,935,788 (90.9%) 121,814,874 (91.4%)	109,002,292 (89.3%) 119,566,626 (89.7%)	15.8 17.2

These samples have already been used in the genome scan carried out in Jaquiéry et al. (2014). The reproductive mode (CP for cyclical parthenogenesis and OP for obligate parthenogenesis) as well as information on Illumina sequencing and read mapping are shown (values in brackets represent the percentage of the total number of pairs sequenced).

### A Major Genomic Region is Associated With Reproductive Mode Variation

**Fig. 1.**
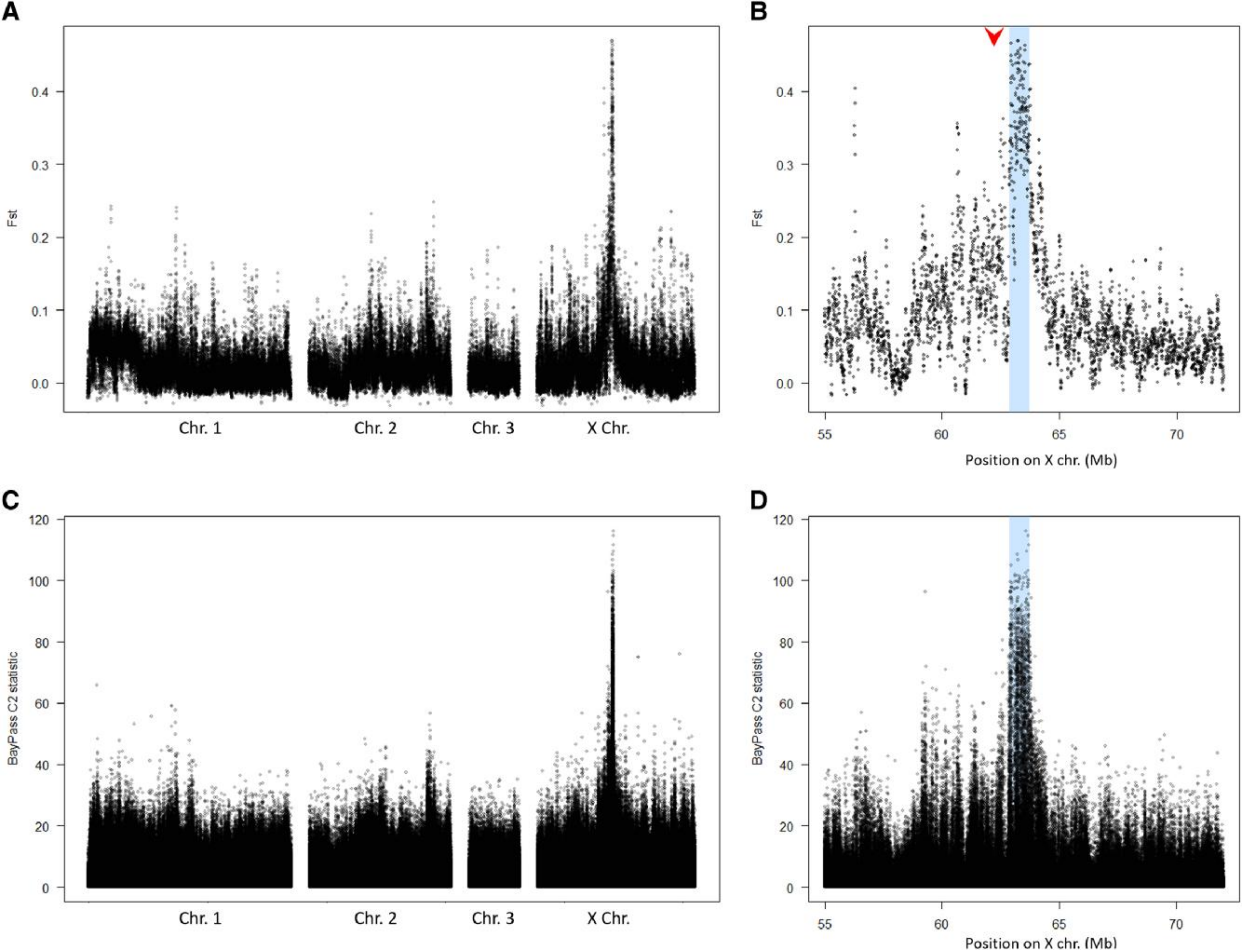
Detection of genomic regions associated with reproductive mode variation in *Acyrthosiphon pisum.* (*A*) Genetic differentiation (*F_ST_*) between OP and CP populations (20-kb windows sliding by 5-kb steps). (*B*) Detail of the region of the X chromosome that contains the main outlier region. The arrow corresponds to the position of the outlier markers (in *F_ST_* scan and QTL analysis) identified in Jaquiéry et al. (2014). (*C*) *C2* statistic from BayPass for each SNP for the whole genome and (*D*) for the main outlier region. The 840-kb region associated with reproductive mode variation is shown in blue.

To identify regions possibly associated with reproductive mode variation, we investigated whether some genomic regions showed unusually high *F_ST_* between OP and CP populations. We also ran BayPass (Olazcuaga et al. 2020), which aims at identifying SNPs associated with a binary trait (here OP or CP reproductive mode) while considering population structure, using the *C2* statistic. Visual inspection of the *C2* statistic and 20-kb sliding windows of *F_ST_* along chromosomes revealed two genomic regions with high *F_ST_* and *C2* values (supplementary file 3, Supplementary Material online): a very short one (∼30 kb) on chromosome 1 and a larger one on the X chromosome. However, the short region on chromosome 1 was found to be misplaced in the v3.0 reference genome (Li et al. 2019) and actually locates on the X chromosome 2 Mb away from the region of highest *F_ST_* (supplementary files 4 and 5, Supplementary Material online). Figure 1 therefore shows *F_ST_* and the *C2* statistic along a corrected genome in which the misplaced region has been moved to its true position. All genomic regions with mean *F_ST_* values higher than 0.25 colocalized in the middle of the X chromosome (see fig. 1*A* and *B*). At least six secondary regions present *F_ST_* values comprised between 0.2 and 0.25. The *C2* statistic also provided a strong support for the main peak in the middle of the X chromosome. Of the 871 SNPs with a *C2* value above 60 (corresponding to a highly significant association, *P* < 10^−14^), 867 were located in the middle of the X chromosome. The remaining four SNPs were located in three different regions, none of which overlapped with the six secondary regions of moderate *F_ST_*. The lack of agreement between *F_ST_* and *C2* methods and the low number of SNPs involved make these nine regions much less reliable that the main outlier region regarding their association with reproductive mode.

**Fig. 2.**
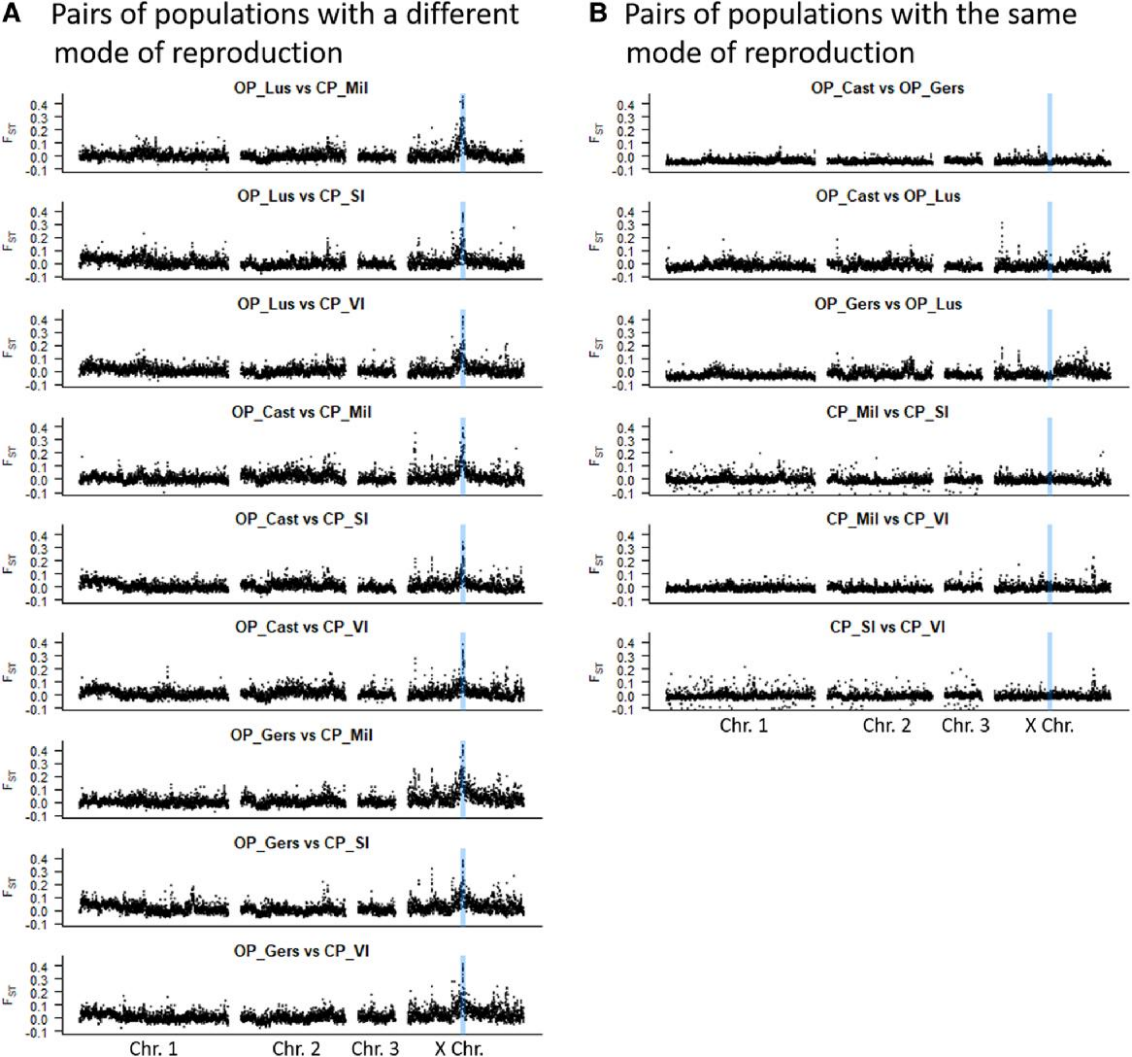
*F_ST_* between pairs of populations in nonoverlapping 100-kb windows sliding along the genome of *Acyrthosiphon pisum*. (*A*) Pairwise comparisons between OP and CP populations. (*B*) Pairwise comparisons between populations with the same reproductive mode. The 840-kb candidate region is shown in blue.

Focusing on the outlier region in the middle of the X chromosome, we observed particularly high *F_ST_* and *C2* values for a ∼840-kb region (highlighted in blue in fig. 1*B* and *D*). This region is flanked by others showing slightly higher values than the rest of the genome, suggesting hitchhiking effect. However, none of these flanking regions was supported by both approaches: for example, the region near position 56 Mb (fig. 1*B*) contains an unusually low number of SNPs (see supplementary file 6, Supplementary Material online), none of which showed significant association with the reproductive mode according to the BayPass *C2* statistic. Based on these results, an 840-kb region from position 62,895,000 to 63,735,000 on the X chromosome, that contained many windows with *F_ST_* above 0.4 and 794 out of 871 SNPs with a *C2* value above 60, was arbitrarily delimited as the main candidate region (fig. 1*A* and *C*). Importantly, this region locates at only 750 kb from the microsatellite marker having the strongest association with reproductive mode variation in QTLs and genome scan analyses using a low density of markers (Jaquiéry et al. 2014). This 840-kb region is highly divergent between CP and OP populations (average *F_ST_* = 0.349) and contains 1,843 SNPs and 240 indels with *F_ST_* > 0.5 between OP and CP populations, a value that denotes very different allelic frequencies between these two population types. This 840-kb region also showed elevated differentiation in every pair of populations differing in their reproductive mode, whereas no such pattern appeared for any pair of populations with the same reproductive mode (fig. 2). No other region showed consistent high differentiation between pairs of populations with different reproductive modes (fig. 2), further supporting a main region associated with reproductive mode variation.

**Fig. 3.**
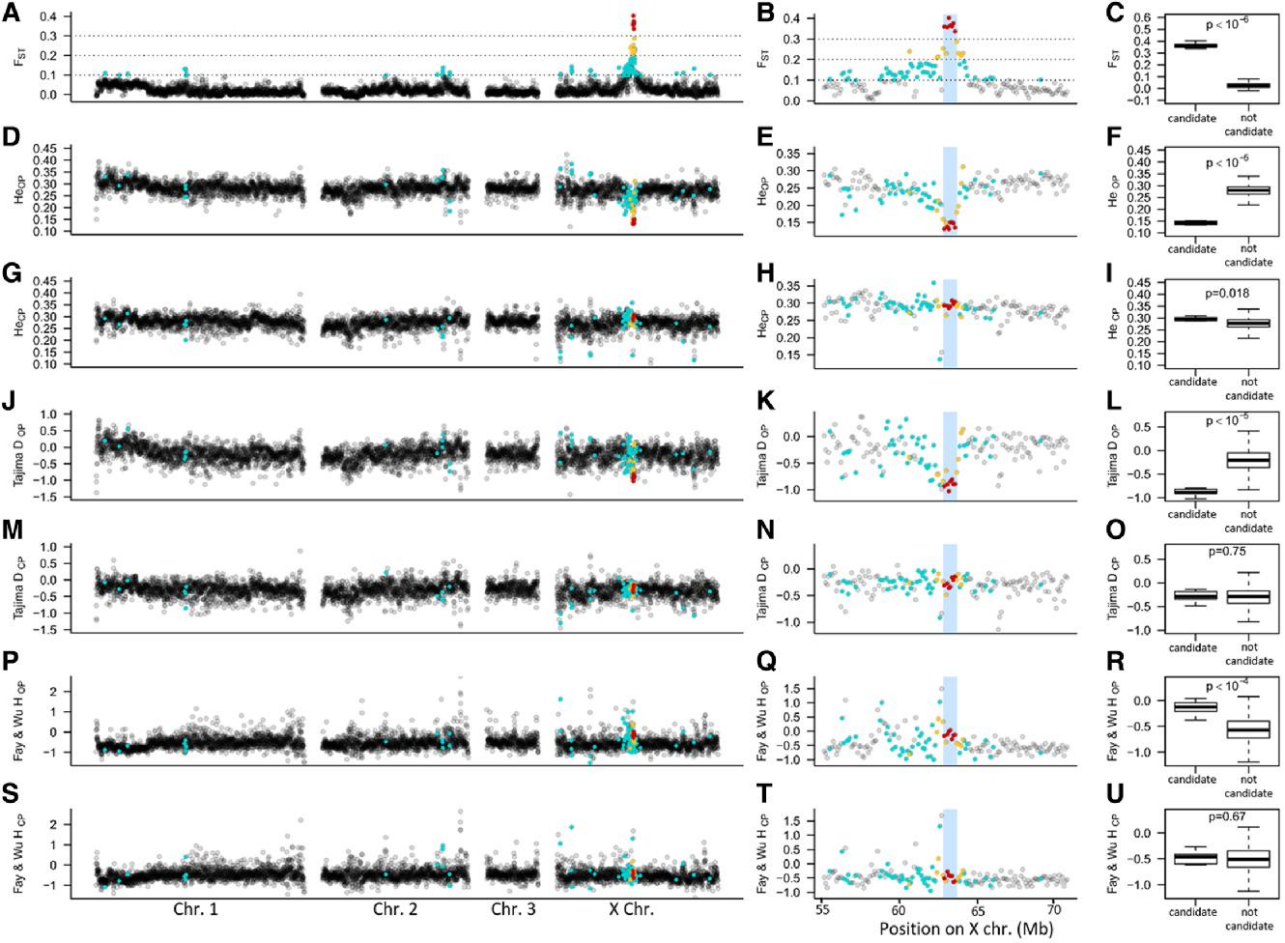
Population genetic indices calculated along the chromosomes of *Acyrthosiphon pisum* in nonoverlapping 100-kb windows. The plots in the first column (panels *A*, *D*, *G*, *J*, *M*, *P*, and *S*) show these indices for the whole genome, those in the second column for the part of the X chromosome carrying the main outlier region (highlighted in blue), and the boxplots in the third column compare the value of the eight 100-kb windows contained within the 840-kb candidate region (“candidate”) with that for the rest of the genome (“not candidate”). *P*-values on boxplots were obtained with two-sided Mann–Whitney tests. Panels *A*, *B*, *C*: *F_ST_* between OP and CP populations; panels *D*, *E*, *F* and *G*, *H*, *I* show expected heterozygosity in OP and CP populations, respectively; panels *J*, *K*, *L* and *M*, *N*, *O* show Tajima’s *D* in OP and CP populations, respectively, and panels *P*, *Q*, *R* and *S*, *T*, *U* show Fay and Wu’s *H* for OP and CP populations, respectively. Each point (a 100-kb window) was colored according to its average *F_ST_* value (red for windows with an average *F_ST_* above 0.3, yellow for *F_ST_* between 0.2 and 0.3, blue for those with *F_ST_* between 0.1 and 0.2, gray for those below 0.1) in order to facilitate the visualization of the possible relationships between regions with high *F_ST_* values and their values at other indices.

To investigate the selection regimes acting on OP and CP populations, heterozygosity, Tajima’s *D* and Fay and Wu’s *H* (*FWH*) were measured per reproductive mode using 100-kb windows along the genome (fig. 3). Genome-wide median heterozygosities were close in the OP and CP populations (*H_E_*_OP_ = 0.280 and *H_E_*_CP_ = 0.276) though they differed significantly (*W* = 6,623,500, *P* < 10^−15^, two-sided Wilcoxon test). Heterozygosity in the 840-kb candidate region was significantly reduced compared with the rest of the genome in OP populations (*H_E_*_OP out_ = 0.14, *U* = 15, *P* = 1.01 × 10^−06^, two-sided Mann–Whitney test, fig. 3*F*), lying within the lower 0.06th percentile of the distribution. Contrastingly, heterozygosity in CP populations (*H_E_*_CP out_ = 0.293) was significantly higher than genome-wide median heterozygosity (*U* = 27,589, *P* = 0.018, fig. 3*I*). Other X-linked regions with moderately high *F_ST_* values (from 0.1 to 0.15) showed reduced diversity in CP but not in OP populations (fig. 3*G*). Genome-wide median Tajima’s *D* were significantly higher in OP than in CP populations (Tajima’s *D*_OP_ = −0.208, Tajima’s *D*_CP_ = −0.292, *W* = 8,578,500, *P* < 10^−15^, two-sided Wilcoxon test). The 840-kb region also stood out in OP populations as being characterized by Tajima’s *D* values (Tajima’s *D*_OP out_ = −0.890) being in the lower 0.8th percentile of the genome distribution. These values are significantly lower than those measured in the rest of the genome (*U* = 503, *P* < 10^−05^, two-sided Mann–Whitney test, fig. 3*L*). No reduction in Tajima’s *D* values was observed in the candidate region in CP populations compared to the rest of the genome (Tajima’s *D*_CP out_ = −0.287, *U* = 19,685, *P* = 0.75, two-sided Mann–Whitney test, fig. 3*O*). To characterize the selective regimes that may have reduced the genetic diversity in OP populations, we measured *FWH*. We observed no reduction of *FWH* in the candidate region in OP populations, the values being actually significantly higher than the genome-wide median (*FWH*_OP out_ = −0.122, *FWH*_OP_ = −0.570, *U* = 33,484, *P* < 10^−4^, fig. 3*R*), providing no support for a hard sweep (Garud et al. 2021). No difference was observed in CP populations (*FWH*_CP out_ = −0.458 and *FWH*_CP_ = −0.508, *U* = 20,187, *P* = 0.67, fig. 3*U*). These patterns suggest selection against lineages not carrying the *op* allele(s) in mild winter regions, leading to high *F_ST_* between reproductive modes and reduced *H_E_* and Tajima’s *D* in OP only in the candidate region. However, the absence of negative *FWH* does not suggest a rapid selective sweep of the *op* allele(s). Interestingly, a 200-kb region located at the left of the 840-kb candidate region also stood out (fig. 3, see also supplementary file 7, Supplementary Material online). It however showed similar patterns in OP and CP populations, including low Tajima’s *D* and *H_E_*, high *FWH*, and low-to-moderate *F_ST_*. We could then assign this region to a previously identified locus associated with wing polymorphism in males of the pea aphid (Braendle et al. 2005; Li et al. 2020), which may be subject to different selective pressures.

**Fig. 4.**
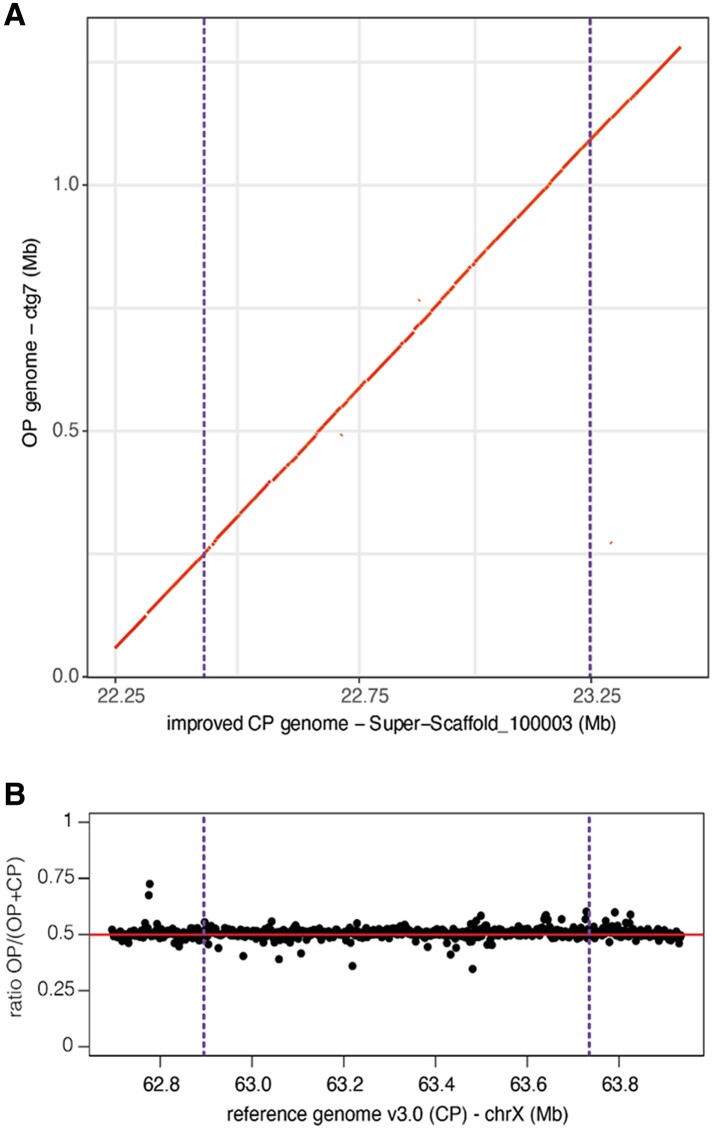
Structure of the 840-kb candidate region in OP and CP genome assemblies. (*A*) MUMmer alignment plot comparing parts of the two contigs (one per genome) containing the candidate region. (*B*) Normalized sequencing depth ratio OP/(OP + CP) calculated over 2-kb windows along the candidate region. In both panels, the vertical dashed lines delimit the 840-kb candidate region.

To better characterize the genetic variation linked to reproductive mode, we investigated the structure of the 840-kb candidate region in the OP and CP genomes. For this, we assembled the genomes of two clones (the OP X6-2 and the CP LSR1 lineages, see supplementary file 4, Supplementary Material online for assembly quality metrics) from long-read sequences. The candidate region was assembled in a single contig in both clones (fig. 4*A*, supplementary file 5, Supplementary Material online) and did not show any large structural rearrangement between these two individual genomes. The sequencing depth ratio OP/(OP + CP) computed over 2-kb windows from Pool-seq data (fig. 4*B*) also failed to reveal any large deletion in OP populations.

### Age of Divergence of the *op* and *cp* Alleles

To approximate the age of the divergence of the *op* and *cp* alleles, we used three different approaches, as none of them was free of bias. The Nei and Li (1979) method based on absolute divergence (*D_a_*, see eq. 1) and the experimentally estimated mutation rate in *A. pisum* (Fazalova and Nevado 2020) indicated a divergence time estimate of 183,129 years (95% CI: 130,513–281,076). The second approach relied on the number of substitutions between *op* and *cp* consensus sequences reconstructed from the Pool-seq data (*N*_mutated sites_) and the same estimated mutation rate (eq. 2). *N*_mutated sites_ was 4,442 (95% CI: 4,340–4,530), such that the divergence time of the *cp* and *op* alleles was estimated to be 555,719 years (95% CI: 388,820–873,824). The third method relied on synonymous divergence (dS) calculated between *op* and *cp* alleles at the 32 genes in the candidate region based on the Pool-seq data. The *dS* was 0.00531 (95% CI: 0.00378–0.00676) and would result in a divergence time estimate of 515,437 years (95% CI: 366,734–656,212) based on the calibrated *dS* between *A. pisum* and *Myzus persicae* (Johnson et al. 2018; Mathers et al. 2020). Similar estimates were obtained when *dS* was measured between the resequenced genomes of an OP (LL01) and a CP individual (L9Ms03) (*dS* = 0.0057, 95% CI: 0.0049–0.0068, *T* = 556,029, 95% CI: 476,440–656,923).

### Gene Content of the Candidate Region

The 840-kb candidate region associated with reproductive mode variation contains 32 predicted genes (table 2). Ten of these showed no homology with *Drosophila* proteins, nine of which were annotated as uncharacterized protein on NCBI, and one (LOC100159148) had homologies with a nuclear pore complex protein from *Salmo trutta* (table 2 and supplementary file 8, Supplementary Material online). The remaining 22 genes have *Drosophila* homologs, including seven that encode proteins of unknown function and 15 that are homologous to *Drosophila* genes with functional annotations and phenotypic characterizations. Interestingly, the amino acid sequences of these 15 genes all share the typical conserved protein domains identified in *Drosophila*, thus giving strong confidence in their annotation (supplementary file 8, Supplementary Material online). More precisely, four are annotated as transcription factors, three of them sharing typical features of zinc-finger proteins (LOC100159233, LOC100161275, LOC107882169). Seven genes are homologous to genes coding for enzymes known to be involved in general metabolism in *Drosophila*: a trimethylguanosine synthase (LOC100570687), a sphingomyelin phosphodiesterase (LOC100169137), an N-acetylglucosaminyltransferase (LOC100569179), a protein kinase (LOC100161186), a fatty acyl-coA reductase (LOC100169017), a Rho GTPase activating protein (LOC100163133), and a cysteine-type peptidase (LOC100163837). Finally, the four remaining genes are homologous to *Drosophila* genes for which phenotypic analyses of mutants revealed their involvement in key biological processes associated with germline and embryo development, including miRNA processing and RNA interference for *Cpb20* (LOC100570523) and *pasha* (LOC100168027), cell cycle control for *APC10* (LOC100165999), and dopamine signaling for *punch* (LOC100164133).

**Table 2:** Annotation of the 32 Genes Predicted in the 840-kb Candidate Region Associated With Reproductive Mode in the pea aphid *Acyrthosiphon pisum*, and Numbers of Nonsynonymous Variants of Different Types Among Cyclical Parthenogenetic (CP) and Obligate Parthenogenetic (OP) Populations

Gene ID	NCBI gene description	*Drosophila* best hit	Annotation in *Drosophila*	Nonsense variants	Frameshift variants	Missense variants	Conservative in-frame deletions	% of positions with depth ≥ 20 in CP	% of positions with depth ≥ 20 in OP
LOC103308741	Uncharacterized protein	CG16854	Uncharacterized protein			1^a^	1	90	90
LOC100570325	Uncharacterized protein	—	Uncharacterized protein			1		100	100
LOC100160994	Alpha-tocopherol transfer protein	CG10026	Uncharacterized protein					100	84
LOC100570523	Nuclear cap-binding protein subunit 2-like	*Cbp20*	Cap-binding protein			1		100	100
LOC100570687	Uncharacterized protein	*Tgs1*	Trimethylguanosine synthase 1					100	100
LOC100169137	Sphingomyelin phosphodiesterase 4	CG6962	Sphingomyelin phosphodiesterase			2^b^		100	100
LOC100569418	Uncharacterized protein	—	Uncharacterized protein					100	100
LOC100569269	Uncharacterized protein	—	Uncharacterized protein					100	100
LOC100569179	UDP-N-acetylglucosamine	*sxc*	N-acetylglucosaminyltransferase					100	100
LOC103308943	Uncharacterized protein	—	Uncharacterized protein					100	100
LOC100163229	Putative nuclease HARBI1	CG43088	Uncharacterized protein					73	78
LOC107883347	Uncharacterized protein	CG4404	Uncharacterized protein					82	82
LOC100161186	MAPK/MAK/MRK overlapping kinase-like	CG42366	Mitogen-activated protein kinase					100	100
LOC100159148	Nuclear pore glycoprotein p62-like	—	Uncharacterized protein					89	87
LOC100168027	Microprocessor complex subunit DGCR8-like	*pasha*	Partner of drosha					100	99
LOC100165999	Anaphase-promoting complex subunit 10	*APC10*	Anaphase-promoting complex					100	100
LOC100568829	Uncharacterized protein	—	Uncharacterized protein			17		100	99
LOC100570789	Uncharacterized protein	—	Uncharacterized protein					100	100
LOC100568498	Uncharacterized protein	—	Uncharacterized protein					98	100
LOC100568585	Uncharacterized protein	—	Uncharacterized protein					100	100
LOC100168655	Scavenger receptor class B member 1	CG40006	Uncharacterized protein					100	100
LOC100169017	Fatty acyl-CoA reductase 1	CG1441	fatty acyl-CoA reductase 1			2		100	100
LOC100163133	Uncharacterized protein	*RhoGAP102A*	Rho GTPase activating protein			1		99	100
LOC100159717	Transcription factor glial cells missing-like	*gcm*	Glial cells missing					98	72
LOC100163837	Bleomycin hydrolase-like	CG1440	Cysteine-type peptidase					100	100
LOC100573568	uncharacterized protein	—	Uncharacterized protein			1		99	79
LOC100573386	Uncharacterized protein KIAA1841 homolog	CG6761	Uncharacterized protein					100	100
LOC100164133	GTP cyclohydrolase 1-like	*Punch*	GTP cyclohydrolase					100	100
LOC100167415	tigger transposable element-derived protein 4-like	*Cag*	Uncharacterized protein			2		43	42
LOC100159233	Zinc-finger protein 180-like	*Crol*	Zn finger protein	1		6		83	80
LOC100161275	Zinc-finger protein 271-like	*Crol*	Zn finger protein					100	100
LOC107882169	Zinc-finger protein 239-like	*Glass*	Zn finger protein		1	1		100	100

a The missense variant is localized in a DUF229 domain.

b The two missense variants are localized in a Mit_SMPDase domain.

Among variants of the candidate region that showed large differences in allele frequencies between OP and CP populations (*F_ST_* > 0.5), 38 impacted protein sequences (table 2 and supplementary file 8, Supplementary Material online). These included 35 missense variants, 1 frameshift variant, 1 conservative in-frame indel, and 1 nonsense variant (table 2 and supplementary file 8, Supplementary Material online), affecting a total of 11 genes. Five of these are homologous to genes encoding uncharacterized proteins. Three genes with homologs in *Drosophila*—*Cbp20* (LOC100570523), *Fatty acyl-CoA reductase* (LOC100169017), and *RhoGAP102A* (LOC100163133)*—*display one or two nonsynonymous SNPs outside the typical functional domains of these proteins. Interestingly, LOC100169137—homologous to a sphingomyelin phosphodiesterase—displays two SNPs in its mit_SMPDase domain, both changing the chemical property of the corresponding amino acid. Finally, two genes sharing features of zinc-finger transcription factors (LOC100159233 and LOC107882169) show polymorphism possibly resulting in truncated proteins in OP lineages. The remaining 21 genes of the region do not display any polymorphism changing the protein sequence between OP and CP lineages.

There was no clear evidence for large indels associated with reproductive mode variation within the 32 genes of the candidate region, as most (29) showed similar sequencing depth in OP and CP populations (table 2 and supplementary file 9, Supplementary Material online). For each of the five genes that had less than 90% of their length sufficiently sequenced in OP and CP populations (LOC100163229, LOC107883347, LOC100159148, LOC100167415, and LOC100159233; table 2), the same gene segment shows reduced sequencing depth in both types of populations (supplementary file 9, Supplementary Material online). For three genes (LOC100160994, LOC100159717, and LOC100573568), the percentage of gene length with sufficient sequencing depth was lower in OP than in CP populations. However, this difference was supported by only one population (out of six) in which the sequencing depth did not meet our criteria, hence failing to indicate consistent lack of coverage in all OP populations.

## Discussion

In this study, we took advantage of a newly available genome assembled at the chromosomal level (Li et al. 2019) to precisely analyze the genomic differentiation between CP and OP populations of the pea aphid, enabling us to pinpoint a main genomic region associated with reproductive mode variation. This 840-kb candidate region carries 32 predicted genes and contains more than 2,000 SNPs and short indels that show strong differences in allelic frequencies between OP and CP populations, making it difficult to identify a causal gene. Population genetic indices revealed a reduction in diversity in this region of 840 kb only in the OP populations, suggesting that selective events have affected only these populations.

### A Major Region is Associated With Reproductive Polymorphism

The main candidate region associated with reproductive variation was relatively large (840 kb) and contained numerous highly differentiated SNPs throughout. We found little evidence for additional regions contributing to this trait. Indeed, other regions show much weaker association with reproductive mode (lower values of *F_ST_* or *C2*) and none was supported by both *F_ST_* and *C2* statistics. Furthermore, our previous QTL analyses (Jaquiéry et al. 2014) showed a close association between reproductive phenotype and genotype at the microsatellite marker closest to the main 840-kb region identified here. We therefore focused on this main region, recognizing that secondary regions could explain the remaining proportion of variance not explained by the main QTL.

Population genetic indices revealed possible signatures of selection acting on the 840-kb region but only in the OP populations, which seems consistent with the OP phenotype being derived from the CP one (the ancestral state in aphids is CP, Davis 2012). Indeed, indices were highly asymmetric, heterozygosity, and Tajima’s *D* being below the 1% of the lower extreme values in OP but close to the mean in CP populations. Interestingly, the reduced diversity at the candidate region in OP populations suggests that one or a few predominant *op* alleles are now present in wild populations. Diversity patterns are also consistent with the QTL-inferred dominance levels of alleles at this locus (Jaquiéry et al. 2014). Since *op* alleles are recessive, OP lineages are necessarily homozygous (explaining the low *H_E_* in OP), whereas CP individuals can be either homozygous for the *cp* allele or heterozygous (hence the higher than average *H_E_* in CP). Overall, these patterns suggest that strong environmental selection on this trait, which is also supported by the close association between reproductive phenotypes and winter climate in other pea aphid populations (Defendini et al. 2023). Nevertheless, *FWH* provides no support for a hard selective sweep in OP populations. This statistic is powerful for detecting recent selective sweeps, but less effective for detecting soft, recurrent, or older sweeps (Kim and Stephan 2002; Przeworski 2002; Zeng et al. 2006), which could be the case here.

### Origin of Reproductive Polymorphism

The large size of the region with high *F_ST_* between reproductive modes is also intriguing. This pattern could result from a point mutation followed by hitchhiking of linked regions. As said above, a single ancient selective sweep, or repeated sweeps, could have occurred—two conditions in which sweeps are not well detected by *FWH* (Kim and Stephan 2002; Przeworski 2002; Zeng et al. 2006). A reduction in recombination rate in the 840-kb region could also generate a uniform differentiation pattern over a large region. Such reduction may have resulted from a large inversion, a type of structural variation that is frequently involved in different polymorphisms (e.g., Joron et al. 2011; Mérot et al. 2020). However, our analyses based on de novo assemblies of OP and CP genomes do not support this hypothesis. Finally, high genetic differentiation between alleles may result from introgression from a divergent population. Indeed, the pea aphid is a complex of host-associated “races” that are still hybridizing (Peccoud et al. 2009, 2014, 2015). The allele that gives rise to an OP phenotype could already be present in another host race and simply have introgressed into the alfalfa race (the race studied here). Another possibility is that the introgression of a DNA segment from another host race (but not involved in the OP phenotype in that race) has caused an incompatibility in the alfalfa race. It could have disrupted the molecular cascade leading to the production of sexual females, resulting in an OP phenotype only in the alfalfa host race.

Whatever the mechanisms that led to the appearance of this polymorphism, the *op* and *cp* alleles appear to be quite divergent—on the order of 180,000–870,000 years. These estimates vary depending on the approach used and should be interpreted with caution as none of the dating methods used are free from bias. Two of them rely on an experimentally measured mutation rate (Fazalova and Nevado 2020) that did not include the sexual phase, yet meiosis is known to be mutagenic (Arbel-Eden and Simchen 2019). The *D_a_*-based method further assumes no selection, and the *dS*-based method relies heavily on a calibrated estimate of interspecies divergence based on few fossils (Johnson et al. 2018). Interestingly, these estimates of allelic divergence time are almost framed by different estimations of the age of the radiation of the pea aphid complex, which also vary widely, from 18,000 to 47,000 years when using the divergence of the maternally inherited obligate endosymbiont *Buchnera aphidicola* in the host aphid lineages (Peccoud et al. 2009) to 419,000–772,000 years when using nuclear divergence (Fazalova and Nevado 2020). Such a large variation does not allow us to determine whether the *op* allele appeared before or after the radiation of the pea aphid complex. This uncertainty could be clarified by testing whether reproductive mode in other pea aphid host races (which also present OP and CP lineages, Frantz et al. 2006) is controlled by homologous *op* and *cp* alleles or some other genetic basis. However, hybridization between most pea aphid host races (Peccoud et al. 2009, 2014, 2015) may make it difficult to determine whether any shared polymorphism arose before the onset of their divergence.

In any case, the age of the *op* allele is likely to be much older than that of most OP lineages (i.e., clones) carrying it. This is the case in *D. pulex*, where asexual lineages are estimated to be 22 years old on average, while the asexual allele is at least 1,250–187,000 years old (Tucker et al. 2013). In the pea aphid, the rare OP males can transmit their *op* allele if they succeed to mate with CP sexual females. This is most likely to occur in regions with intermediate winter temperature, where both types of lineages are expected to be sympatric. These crosses would allow the *op* allele to escape from linked deleterious mutations that may accumulate in OP lineages, and may also generate new OP lineages, ensuring the long-term persistence of OP populations (and *op* allele) through “contagious asexuality”. This mechanism would thus ensure the persistence of the *op* allele even in the face of recurrent extinctions of OP lineages, leading to higher age of divergence between *op* and *cp* alleles than the age of currently existing OP lineages. This scenario of contagious asexuality is supported by the low genetic differentiation between OP and CP populations (genome-wide average *F_ST_* of 2.5%), which necessary requires some gene flow to homogenize genomes. Although contagious asexuality needs to be tested in nature for the pea aphid, previous works have verified this scenario in natural populations of other aphid species (Halkett et al. 2008). The strong contrast in winter temperatures between western European regions may further facilitate the long-term maintenance of the two alleles, allowing the two population types to persist stably in the areas to which they are adapted (e.g., Defendini et al. 2023).

Interestingly, another locus of considerable ecological importance locates at less than 150 kb of the 840-kb region associated with reproductive mode. This locus, called *aphicarus*, determines wing polymorphism in pea aphid males (Braendle et al. 2005; Li et al. 2020). We observed a specific genetic signature near *aphicarus* (fig. 3 and supplementary file 7, Supplementary Material online), which includes a drop of *F_ST_* between OP and CP populations, a drop of both heterozygosity and Tajima’s *D* in OP and CP populations and an increased *FWH* in both populations. The frequency of the derived wingless allele is high in the alfalfa-adapted host race in Europe (around 90–95%, Frantz et al. 2009; Li et al. 2020), which could explain the low differentiation between OP and CP populations and the low *H_E_* and Tajima’s *D* at this locus. The high values for *FWH* are puzzling, but could be related to balancing selection on wing polymorphism (the two phenotypes coexist in many host races, Li et al. 2019), or to the genotype of the outgroup (winged or wingless). In any case, we observed no association between *aphicarus* and the 840-kb candidate region, with reduction of diversities observed in both OP and CP populations for *aphicarus*, but limited to OP populations for reproductive mode variation. This pattern is compatible with the relative divergence time of the two polymorphisms, the wing polymorphism estimated to be at least six times older on the basis of *dS* (0.0386, Li et al. 2019) than the reproductive polymorphism (∼0.0053–0.0057).

### Functional Insights into the Genetic Determinants of the Loss of Sex

As our analyses did not reveal large structural variation between *cp* and *op* alleles, reproductive mode variation probably involves only small-sized polymorphisms. SNPs and small indels are frequent along the candidate region and may affect reproductive mode by altering the function of genes controlling the switch to the sexual phase. Previous transcriptomic studies have identified a number of genes that change expression during the transition from asexual to sexual reproduction in a CP lineage of the pea aphid (Le Trionnaire et al. 2007, 2008, 2012; Gallot et al. 2012). Interestingly, one of the 32 genes located within the candidate region (*cbp20*) corresponds to a gene differentially expressed between sexual and asexual germlines (Gallot et al. 2012). Its *Drosophila* homolog encodes an mRNA cap-binding protein involved in miRNA processing and gene silencing by RNAi and germline *Drosophila* mutants produce no eggs (Sabin et al. 2009). Remarkably, this gene also showed a nonsynonymous polymorphism with a high *F_ST_* between the OP and CP populations. Although this variation lies outside the typical RNA recognition motif domain of the protein, functional analyses by CRISPR/Cas9 targeted mutagenesis are now required to assess whether this variation determines reproductive mode.

Ten other genes are affected by nonsynonymous polymorphisms between the OP and CP populations. Five are of unknown function and five have a predicted function with no apparent link to variation in reproductive mode. Among the latter are two genes containing zinc-finger domains that are truncated or show a frameshift, probably leading to nonfunctional proteins in OP lineages. However, these two proteins do not share strong similarities with well-characterized *Drosophila* transcription factors, making it difficult to predict the phenotypic consequences of their disruption.

Interestingly, three other genes of the candidate region (*Pasha*, *APC10*, and *punch*) share similarities with *Drosophila* genes whose functions could play a role in reproductive mode switch in aphids. *Pasha* is involved in miRNA biogenesis: germline mutants do not form cysts from the germarium and fail in oocyte fate determination (Azzam et al. 2012). *APC10* promotes metaphase to anaphase transition during the cell cycle, and germline mutants show defects in stem cells production (Liu et al. 2016). Finally, *punch* is involved in eye pigmentation and cell cycle control. Some mutants show defaults in dopamine synthesis and embryo development (Hsouna et al. 2007). None of these three genes contained nonsynonymous variants between the OP and CP populations, but polymorphisms outside protein-coding sequences could also control reproductive mode variation. Indeed, intergenic and intronic regions contain DNA motifs to which regulatory factors may bind. Transcriptomic analyses of OP and CP lineages submitted to long and short photoperiod regimes would allow testing whether some of the genes of the candidate region are differentially expressed, thus whether they could control the reproductive polymorphism through differences in protein levels. A parallel can be drawn with *D. pulex*, where male production is genetically controlled (Innes and Dunbrack 1993; Innes 1997). A recent genome scan analysis pinpointed a single gene whose male-producing and nonmale-producing alleles differ by seven nonsynonymous substitutions (Ye et al. 2019). These alleles are also expressed at different levels in response to the environmental cue normally inducing the production of males (Ye et al. 2019). Whether pea aphid reproductive polymorphism is determined by expression levels, protein variants, or a combination of both remains an open question.

## Conclusion

This work refines the size, location, and gene content of the locus associated with sex loss in the pea aphid. Further functional studies are needed to identify the gene(s) driving reproductive mode variation, and to determine whether variation in this trait depends on variation in protein sequence and/or protein levels. Transcriptomic analyses of OP and CP lineages exposed to long and short photoperiods should help to identify the causal gene(s) and underlying mechanisms. CRISPR/Cas9 targeted mutagenesis, which has been successfully developed in the pea aphid (Le Trionnaire et al. 2019), would then allow a functional validation of the role of candidate genes. Furthermore, exploring the genetic basis of sex loss in other host races and species should clarify whether reproductive mode variation, which is widespread in aphids (Moran 1992; Simon et al. 2002), relies on common or independent mechanisms, and whether introgression might be involved. Finally, sequencing individual OP lineages in various populations would allow assessing the accumulation of deleterious mutations in these clones and whether this factor primarily dictates their fate.

## Materials and Methods

### Aphid Sampling

This study is based on the *A. pisum* samples previously used to conduct a low-density microsatellite-based genome scan (Jaquiéry et al. 2014). Briefly, parthenogenetic females were collected on *Medicago sativa* in alfalfa-cultivated fields from six sampling sites in 2008 (table 1). Three sites located in north-east France and Switzerland, where only CP lineages can survive cold winters. The three other sites located in south-west France where winters are generally mild and therefore favor OP. For each of the six geographical populations, we succeeded to keep alive 14–21 genetically distinct clonal lineages, each initiated by a sampled female (table 1). Clonal individuals for each lineage were collected in 2008 and stored in absolute ethanol at −20 °C until DNA extraction in 2013.

### Pool Sequencing

DNA was extracted from four fourth instar larvae per clonal lineage using the Qiagen DNeasy Blood and Tissue Kit (Qiagen, Hilden, Germany) following the manufacturer’s instructions. After RNAse treatment, DNA solutions were pooled in equimolar proportions for each population ensuring that clonal lineages contributed equivalent amounts of DNA to the pool. Two independent paired-end libraries were constructed per population from these DNA pools using the Genomic DNA Sample Preparation Kit (Illumina, San Diego, CA) (technical replicates). The resulting 12 libraries were sequenced in 2013 on four lanes of the Illumina HiSeq 2000 platform in a single 2 × 100-cycle run using Illumina Sequencing Kit v3, producing between 11.7 and 15.7 million paired-end 100 bp reads per library (table 1). The raw data are publicly available at the Sequence Read Archive of the NCBI database, under the BioProject ID PRJNA454786.

### Mapping

For read mapping, we used the v3.0 reference genome of the pea aphid (NCBI: pea_aphid_22Mar2018_4r6ur, Li et al. 2019). This assembly is 541 Mb in size and consists of four main scaffolds corresponding to the three autosomes and the X chromosome, and 21,915 additional short scaffolds not positioned on chromosomes (which account for 14% of the bases, Li et al. 2019). Paired-end reads were mapped to a fasta file containing the *A. pisum* reference genome v3.0 and the sequences of its known endosymbionts (Guyomar et al. 2018) with bwa-mem v0.7.10 (Li and Durbin 2009), using defaults parameters. Only primary and properly paired alignments were kept with SAMtools v1.6 (Li et al. 2009). Read pairs corresponding to duplicates were then identified with Picard Markduplicates v2.18.2 (http://broadinstitute.github.io/picard/) and removed. The final number of read pairs kept ranged from 10.5 to 14.1 million, resulting in a sequencing depth per library of 15.1–20.4× (see table 1 for mapping statistics).

### Variant Calling

The 12 alignment (BAM) files corresponding to the 12 DNA libraries were merged in a single mpileup file using SAMtools (Li et al. 2009) and a sync file was created using Popoolation2 (Kofler et al. 2011) with default parameters except for a minimum base quality set to 20. Positions corresponding to the aphid symbiont and mitochondria genomes were removed from the sync file, to analyze the pea aphid nuclear genome only.

A total of 11,954,278 SNP positions were identified, in which the least frequent allele was represented by at least four reads (MAC ≥4). This dataset also included 181,204 tri-allelic SNPs for which the third (least frequent) allele was represented by only one read. This third allele was ignored and the SNP was considered biallelic. We checked that this subdataset showed the same signal as the full dataset (supplementary file 10, Supplementary Material online).

To visualize the structure of the dataset and check that replicates from the same population cluster together as expected, a principal component analysis (PCA) was carried out on the 12 libraries with prcomp in R version 3.6.1 (R Core Team 2019). We used allele frequencies at 50,000 randomly drawn SNPs. Since the two libraries from each population grouped together (supplementary file 2, Supplementary Material online), we summed their allele counts as if they constituted only one library in all subsequent analyses. Thereafter, we refer to this dataset of 11,954,278 SNPs as the nonfiltered dataset.

Additional filters were then applied to select reliable and informative SNPs for the *F_ST_* and heterozygosity estimation. First, only SNP positions with a sequencing depth higher than 20 and lower than 60 per population were considered, the mean depth ranging from 31 to 37 depending on the population. The upper limit of 60 was chosen to avoid duplicated genomic regions not resolved in the reference genome, and the lower limit of 20 to discard SNPs whose sampling was too low for reliable allele frequency estimates. Second, a minor allele frequency threshold of 5% was applied to eliminate SNPs harboring rare alleles and which are not informative for a *F_ST_*-based genome scan. After applying these selection criteria, we obtained a filtered dataset of 4,633,747 SNPs.

### Detection of Genomic Regions Associated With Reproductive Mode Variation

We ran BayPass under the core model using the nonfiltered SNP dataset to compute the *C2* genetic differentiation statistic (Olazcuaga et al. 2020). This *C2* statistic compares the standardized population allele frequencies (i.e., the allele frequencies corrected for the population structure) between the two groups of populations specified by the binary covariable of interest, here the reproductive mode (CP or OP). As the number of SNPs was very large and computing time increases nonlinearly with the number of SNPs, we divided the full dataset into 100 subdatasets containing only 1 SNP every 100 SNPs along the genome as recommended by Gautier et al. (2018). This strategy allowed analyzing all available SNPs while limiting the influence of linkage disequilibrium. After checking for convergence with three independent runs of the first subdataset using options -nval 500 -thin 200 -npilot 10 -pilotlength 300 -burnin 500 for the Markov chain Monte Carlo algorithm, we ran the analysis on all 100 subsets.

In parallel, to estimate genetic differentiation (*F_ST_*) between populations with different reproductive modes, we summed allele counts in the filtered dataset (4.6 million SNPs) for the three CP populations on one hand and for the three OP populations on the other hand. These counts were used to calculate *F_ST_* at each SNP between reproductive modes with the R package poolfstat, which implements *F_ST_* estimates for Pool-seq data (Hivert et al. 2018). We then calculated the average *F_ST_* within 20-kb windows sliding by 5-kb steps to smooth its variation along the genome and precisely identify regions of high differentiation. *F_ST_* within nonoverlapping 100-kb windows between the two reproductive mode and between all possible pairs of populations were computed in the same fashion.

Heterozygosity (*H_E_*, following Nei 1973) was calculated per type of populations (OP or CP) at each SNP using allele frequencies from the filtered dataset. The mean *H_E_* per population type was then computed in 100-kb contiguous windows. To detect potential selective sweeps, Tajima’s *D* and *FWH* were calculated for each population type. For Tajima’s *D*, we used the pileup-formatted SNP files for the pool samples of each reproductive mode (i.e., one OP and one CP population) generated previously. We randomly subsampled the datasets as recommended to achieve a uniform depth using PoPoolation 1.2.2 (Kofler et al. 2011), using the following parameters: –target-coverage 30 –max-coverage 360 –min-qual 20. Tajima’s *D* was then calculated using PoPoolation 1.2.2 over 100-kb nonoverlapping windows with the following parameters: –min-count 2 –min-covered-fraction 0.5. Then, *FWH* was calculated with npstat (Ferretti et al. 2013) for each type of population (OP or CP) on 100-kb windows. Input files consisted of the pileup-formatted SNP files for the pool samples of each reproductive mode, and a fasta file of an outgroup (here, a cryptic species of the pea aphid complex adapted to feed on *Ononis spinosa*, Peccoud et al. 2009). For this, Illumina pair-end 100 bp whole-genome re-sequencing data of a CP *A. pisum* individual of the *Ononis* host race used in a previous study (Guyomar et al. 2018) was retrieved from NCBI (Project PRJNA255937—SRX661218). After filtering the reads with fastp v0.20, reads were aligned with bwa-mem v0.7.17 on the v3.0 reference genome of the pea aphid with default parameters, and duplicates were marked with GATK MarkDuplicates v4.1.4.1. SNP variants were called with GATK HaplotypeCaller v.4.1.1 with options –heterozygosity 0.001 –heterozygosity-stdev 0.01 –sample-ploidy 2. SNP variants with a GQ > 20 were then used to modify the v3.0 genome sequence to produce a fasta sequence for this outgroup. Positions homozygous for the alternative allele were replaced by the alternative allele, and for those identified as heterozygous, alleles were drawn at random, with equiprobability (using the R function rbinom). We used the following parameters to run npstat: -l 100000 -mincov 4 -maxcov 180 -minqual 20 -nolowfreq 3.

Differences in *H_E_*, Tajima’s *D* and *FWH* between the OP and CP populations were evaluated with two-sided Wilcoxon tests (using 100-kb nonoverlapping windows as the statistical unit). Within each reproductive mode, two-sided Mann–Whitney tests were used to test for differences in these population genetic indices between candidate region(s) for reproductive mode variation and noncandidate regions. Note that the lack of independence between windows that are physically linked within the same candidate region may pose an issue for these tests.

### Comparison of the Structure of the Candidate Region in CP and OP Genomes

The above analyses identified a genomic region as main candidate to explain the variation in reproductive mode. To compare the structure of the candidate region between CP and OP genomes, we assembled the genome of an OP lineage (clone X6-2, Jaquiéry et al. 2014), as the *A. pisum* reference genome v3.0 (Li et al. 2019) was assembled from a CP lineage (clone LSR1, IAGC 2010). Oxford Nanopore technology was used to obtain long-read sequences from the OP lineage and to build a de novo genome assembly (see supplementary file 4, Supplementary Material online for details). We also found that the *A. pisum* reference genome v3.0 (Li et al. 2019) contained some small assembly errors which could impact our results (see Results section and supplementary files 4 and 5, Supplementary Material online). We therefore constructed a new assembly for the LSR1-CP lineage (referred to as “improved CP genome” hereafter) with ONT- and PacBio-generated long reads and optical map data (supplementary file 4, Supplementary Material online). We then compared the structure of genomes assemblies at a 1.25-Mb region containing the 840-kb candidate region using MUMmer v3.22 (Kurtz et al. 2004). Pairwise alignments of the CP and OP genome sequences were assessed using NUCmer v3.07. Results were filtered using the delta-filter script to keep optimal correspondence with a minimum length of 1,000 bp and a minimum alignment identity of 90%, and were visualized using MUMmerplot v3.5 (Kurtz et al. 2004). Complementarily, to investigate the possible deletion of short genomic regions in OP populations, we plotted the sequencing depth ratio OP/(OP + CP) from the Pool-seq data on the v3.0 genome sequence. The sequencing depths of the OP and CP populations were normalized prior to ratio calculation, so that a ratio of 0.5 is expected for genome segments presenting the same copy number in the OP and CP populations. To visualize results, we computed the average of this ratio over 2-kb nonoverlapping windows on the candidate region.

### Age of Divergence of *op* and *cp* Alleles

To roughly estimate the divergence time between the *op* and *cp* alleles of the main candidate region, we used three different approaches, each with its own limitations. The first two used the complete DNA segment of the candidate region (coding and noncoding sequences), while the third one used coding sequences only. Divergence time (*T*) was first estimated as


(1) $$T=D_{a}/(2\times \mu ),$$

where *D_a_* is the absolute divergence at the candidate region and *µ* is the substitution rate (Nei and Li 1979). A related estimate of *T* was obtained as


(2) $$T=N_{\mathrm{mutated}\;\mathrm{sites}}/(2\times N_{\mathrm{sites}}\times \mu ),$$

where *N*_mutated sites_ is the number of observed substitutions between the two alleles over all callable sites *N*_sites_. The third approach relied on the *dS* between *op* and *cp* alleles at the 32 genes from the candidate region and the *dS* between two aphid species of known divergence time to infer *T* assuming proportionality.

For the first approach (eq. 1), *D_a_* was estimated from the allele frequencies in the nonfiltered Pool-seq data, as in Cruickshank and Hahn (2014). A mutation accumulation experiment in the pea aphid estimated the mutation rate *µ*_parth_ to 2.7 × 10^−10^ (95% CI: 1.9 × 10^−10^–3.5 × 10^−10^) per parthenogenetic generation (Fazalova and Nevado 2020). The annual mutation rate for an OP lineage was thus estimated as *µ*_op_ = *N*_gen_ × *µ*_parth_, *N*_gen_ being the number of generations per year (estimated to 15). For a CP lineage, we followed Fazalova and Nevado (2020) and estimated the mutation rate as *µ*_cp_ = (*N*_gen_−1) × *µ*_parth_ + *µ*_sex_, where *µ*_sex_ (2.96 × 10^−9^; 95% CI: 1.52 × 10^−9^–4.99 × 10^−9^) is the average mutation rate per sexual generation in insects (Keightley et al. 2014, 2015; Yang et al. 2015; Liu et al. 2017; Oppold and Pfenninger 2017) as there is no such estimate for aphids. The mutation rate to consider in equation (1) was thus 5.39 × 10^−9^ (95% CI: 3.52 × 10^−9^–7.57 × 10^−9^).

For the second approach (eq. 2), in addition to the mutation rate (obtained above), we had to measure the number of substitutions between the two alleles, hence to reconstruct *op* and *cp* consensus sequences. For each reproductive mode, we randomly drawn alleles at polymorphic SNPs in the 840-kb region, where the probability of being drawn equals the allele frequency (using the R function rbinom). Then, we counted the number of substitutions between the *op* and *cp* consensus sequences (i.e. *N*_mutated sites_). This step was repeated 100 times to get 95% confidence interval for *N*_mutated sites_. *N*_sites_ was determined as the number of callable sites with sequencing depth ≥20 in every population (740,918).

For the third approach, coding sequences were extracted from each replicate of the consensus *op* and *cp* sequences generated above, and median *dS* between *op* and *cp* alleles at the 32 genes from the candidate region were computed with the seqinr R package (Charif and Lobry 2007). This step was repeated on the 100 replicates of consensus sequences to get 95% CI for *dS*. Divergence time was then estimated by proportionality, assuming constant synonymous mutation rate between *cp* and *op* alleles and between the pea aphid and the peach–potato aphid *M. persicae*, whose divergence is estimated at some 22 million years ago and corresponds to a *dS* of 0.2268 (Johnson et al. 2018; Mathers et al. 2020). *dS* at the 32 genes were also estimated using the genomes of two individuals of the alfalfa host race, which were previously sequenced using 100 bp Illumina read pairs. One is of CP phenotype (clone L9Ms03—Project PRJNA255937—SRX661210, see Guyomar et al. 2018) and the other of OP phenotype (clone LL01—PRJNA255937—SRX20811676). Read filtering, mapping, SNPs calling and the construction of consensus sequences were carried out as previously described for the resequenced genome of an individual from the *O. spinosa* host race (see the nspstat analyses), and *dS* between *op* and *cp* alleles at the 32 genes computed with seqinR. A total of 100 replicates of the consensus sequences were built to calculate 95% CI, since these sequenced individuals were heterozygous at some SNPs.

### Gene and Variant Annotation in the Main Candidate Region

Amino acid sequences of the predicted genes present in the 840-kb candidate region were retrieved from the v3.0 version of the pea aphid genome assembly. Annotations for these genes were obtained from the general feature format (gff) file available on NCBI (GCF_005508785.1_pea_aphid_22Mar2018_4r6ur_genomic.gff.gz). Whenever a gene had multiple predicted transcripts, we only kept the longest transcript. A BlastP analysis (Altschul et al. 1990) was then performed against Flybase (http://flybase.org/) to identify the closest *Drosophila* homolog for each of these aphid genes (at *P* < 10^−7^). Conserved protein domains were identified and annotated for each gene using the SMART web resources (http://smart.embl-heidelberg.de/; Letunic et al. 2020) with the “normal” mode and a significance level of 10^−10^. To detect potentially causal polymorphisms, we examined the variants (SNPs and short indels) from the candidate region for reproductive mode variation showing *F_ST_* above 0.5 and a sequencing depth ≥ 20 in every population. These variants were classified according to their impact on gene structure by SnpEff v4.3t (Cingolani et al. 2012) with default parameters and using the GFF file available on NCBI. Variants with moderate-to-high predicted impact were retained for further analysis. This includes variants resulting in premature stop codons, frameshifts, missenses, or conservative in-frame indels.

## Supplementary Material

evad168_Supplementary_Data

## Data Availability

Raw sequence reads are deposited on NCBI (PRJNA454786, PRJNA745262, PRJNA255937-samples SRX661210, SRX20811676, and SRX661218). Genome assemblies are available at the following permanent addresses: https://bipaa.genouest.org/sp/acyrthosiphon_pisum/download/genome/LSR1_CP/; https://bipaa.genouest.org/sp/acyrthosiphon_pisum/download/genome/OP/. The data and scripts are available on Zenodo: doi.org/10.5281/zenodo.8116727.
