# Supplementary material for: Contrasting Evolutionary Patterns Between Sexual and Asexual Lineages in a Genomic Region Linked to Reproductive Mode Variation in the pea aphid
Source: Genome Biol Evol. 2023 Sep 17;15(9):evad168. doi: 10.1093/gbe/evad168 (PMC10538257; doi:10.1093/gbe/evad168)
Supplement: evad168_Supplementary_Data [file evad168_supplementary_data.zip › supplmat_Rimbault_31.08.23.docx]

**Supplementary materials**

### **Supplementary File 1.** Principal component analysis (PCA) score plot of the first two components calculated on allele frequencies for the 12 libraries (six populations, with two replicates each) constructed from *A. pisum* DNA extracts. Only 50,000 randomly drawn SNPs were used. The results remain unchanged when we repeat this analysis using a set of another 50,000 randomly selected SNPs. OP populations are shown in blue and CP populations in red. The first axis (that accounts for 27.5% of the variance) separates populations by reproductive mode and the second axis discriminates mainly the different OP populations (13.5% of variance explained). Details on the populations are given in Table 1 of the main text.


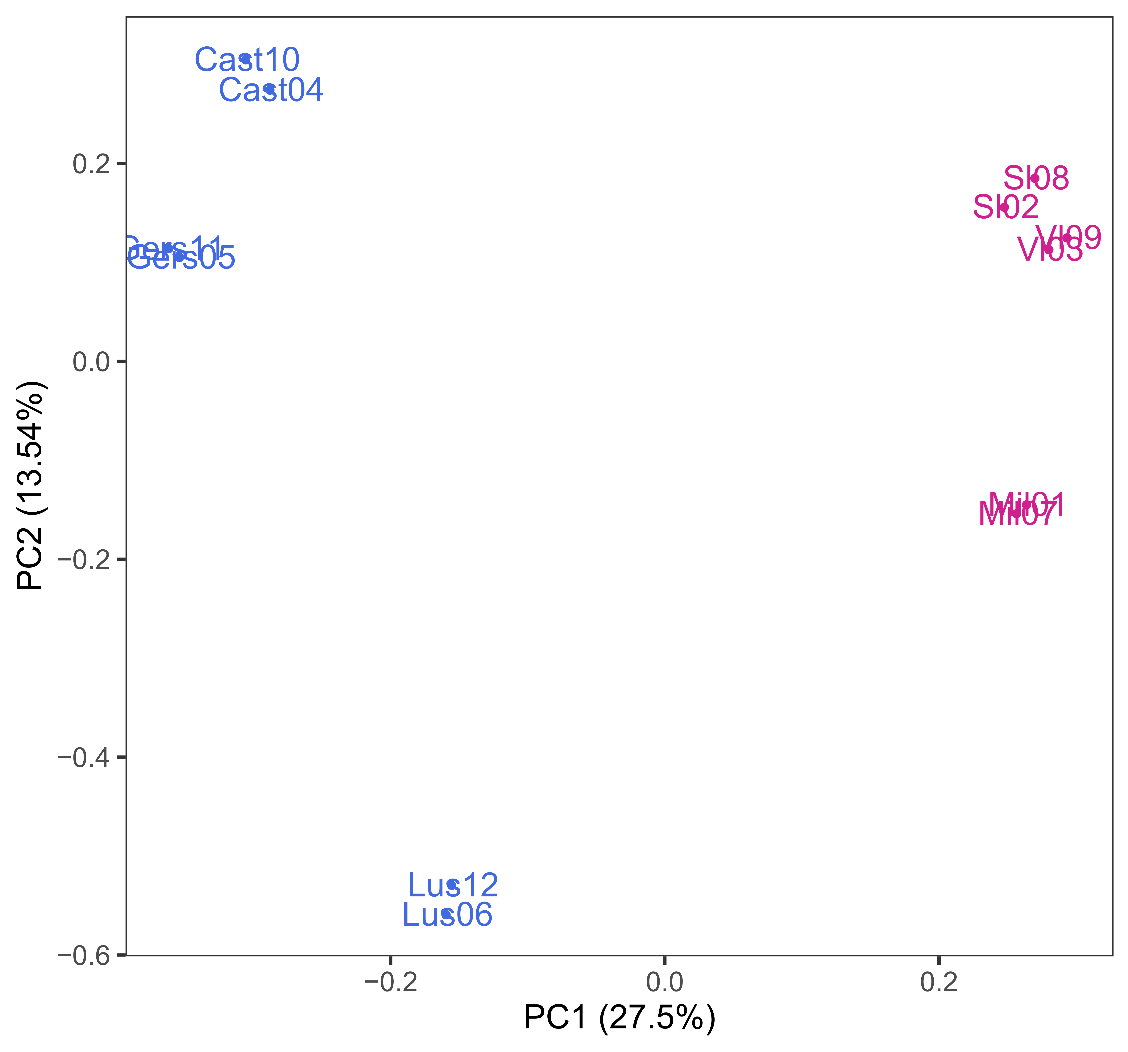


Supplementary File 2. Paiwise *F_ST_* between populations of *A. pisum*. Comparisons between pairs of populations of the same reproductive mode are highlighted in grey. CP: cyclical parthenogenetic populations, OP: obligate parthenogenetic populations. Details on the populations are given in Table 1 of the main text.

|  | OP_Cast | OP_Gers | OP_Lus | CP_Mil | CP_Sl | CP_Vl |
| --- | --- | --- | --- | --- | --- | --- |
| OP_Cast | - | -0.0447 | -0.0197 | 0.0112 | 0.0055 | 0.0104 |
| OP_Gers | - | - | -0.0261 | 0.0138 | 0.0152 | 0.0156 |
| OP_Luz | - | - | - | -0.0006 | 0.0052 | 0.0059 |
| CP_Mil | - | - | - | - | -0.0106 | -0.0118 |
| CP_Sl | - | - | - | - | - | -0.0137 |
| CP_Vl | - | - | - | - | - | - |

### **Supplementary File 3.** Detection of genomic regions associated with reproductive mode variation along the genome of *Acyrthosiphon pisum.* A) Genetic differentiation (*F_ST_*) between obligate parthenogenetic (OP) and cyclical parthenogenetic (CP) populations (20-kb windows sliding by 5-kb steps). B) Detail of the portion of the X chromosome that contains the main outlier region. C) *C2* statistic from BayPass for each SNP for the whole genome and (D) for the main outlier region. The *C2* statistic contrasts allele frequencies between OP and CP populations while correcting for population structure, to identify SNPs associated with this binary trait. The 840-kb region identified as the main candidate is shown in blue. The red arrow identifies a 30 kb region misplaced in the v3.0 genome assembly (Li, et al. 2019), which is actually located at 2 kb from the main outlier region in the middle of the X chromosome. This misassembled region (see also Supplementary Files 4 and 5 for more details) was moved to its expected position in Figure 1.


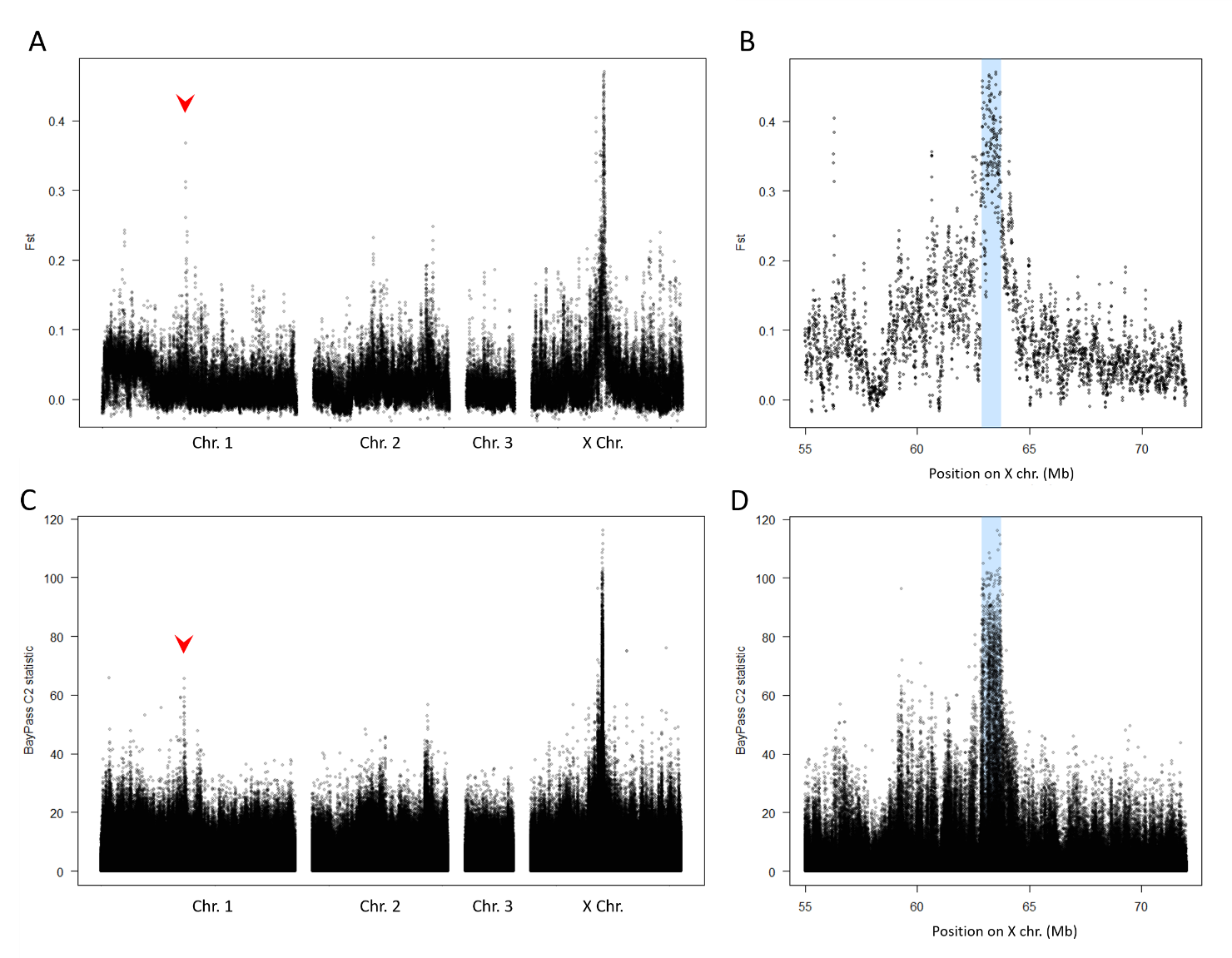


**Supplementary File 4.** This supplementary file describes the new data that were sequenced to build new genome assemblies for the OP and CP lineages of the pea aphid, the construction of these assemblies and the analyses that were performed on the 840-kb candidate region with these genomes.

*OP genome sequencing and assembly*

We sequenced and assembled an OP genome with long reads (Oxford Nanopore, ONT). The lineage selected for sequencing (lineage X6-2 from cross 6 in the Supplementary Figure S1 from Jaquiéry, et al. 2014) was characterized as OP in conditions inducing sexual morph production. DNA was extracted from parthenogenetic embryos dissected from 20 adult females of this X6-2 OP lineage. Embryos were ground in lysis buffer using a potter. The lysis solution was then used in a Phenol protocol using high vacuum grease silicone to separate aqueous and organic phases without pipetting, and low centrifuge speed to preserve DNA integrity. The DNA was directly precipitated with isopropanol and then resuspended in ultra-pure sterilized water. DNA concentration was measured with Qubit fluorometer and DNA fragment length (range 17kb-100kb) was assessed with both a Pulse Field Gel Electrophoresis and a Femto Pulse Run.

A Nanopore genomic DNA library was then prepared using the Ligation Sequencing Kit (Oxford Nanopore Technologies), following the manufacturers protocol, and sequenced on an R9.4 flow cell for 72 hours (ONT GridION technology). See suppl. Table 1 (in the present file) for a summary of the characteristics of sequence data obtained, which are publicly available on NCBI (BioProject ID PRJNA745262).

The Nanopore reads were first trimmed with PoreChop v0.2 (Wick, et al. 2017), using default parameters. Reads were then assembled with wtdbg v2.5 (Ruan and Li 2020) with the options -x ont -g 500m and polished with the same tools after an alignment step of the trimmed reads with minimap v2.14 (Li 2018). The characteristics of this OP genome assembly are summarized in Table 2 (this file). This genome assembly is publicly available at <https://bipaa.genouest.org/sp/acyrthosiphon_pisum/download/genome/OP>/.

*Improved CP genome sequencing and assembly*

As the genome available for a CP lineage of the pea aphid (reference v3.0, Li, et al. 2019) contains small assembly errors – which could have affected our conclusions if the candidate region was involved – we chose to build a new genome assembly for a CP lineage. To do so, we combined PacBio and ONT sequencing data with an optical map performed on a CP lineage of the pea aphid (clone LSR1).

For the optical map, ultra-high molecular weight (uHMW) DNA was purified from 0.2 g of frozen larvae from the LSR1 aphid lineage according to the Bionano Animal Tissue DNA Isolation Grinding Protocol (800002 - Bionano Genomics) with the following specifications and modifications. Briefly, the aphid larvae were disrupted in the homogenization buffer with a potter. Nuclei were washed and then embedded in agarose plugs. After overnight proteinase K digestion in the presence of Lysis Buffer (Bionano Genomics) and one hour treatment with RNAse A (Qiagen), plugs were washed four times in 1x Wash Buffer (Bionano Genomics) and five times in 1x TE Buffer (ThermoFisher Scientific). Then, plugs were melted two minutes at 70°C and solubilized with 2 µL of 0.5 U/µL AGARase enzyme (ThermoFisher Scientific) for 45 minutes at 43°C. A dialysis step was performed in 1x TE Buffer (ThermoFisher Scientific) for 45 minutes to purify DNA from any residues. The DNA samples were quantified using the Qubit dsDNA BR Assay (Invitrogen). Megabase-sized DNA fragments were visualized by pulsed field gel electrophoresis (PFGE).

Labeling and staining of the uHMW DNA were performed according to the Bionano Prep Direct Label and Stain (DLS) protocol (30206 - Bionano Genomics). Briefly, labeling was performed by incubating 750 ng genomic DNA with 1× DLE-1 Enzyme (Bionano Genomics) for two hours in the presence of 1× DL-Green (Bionano Genomics) and 1× DLE-1 Buffer (Bionano Genomics). Following proteinase K digestion and DL-Green cleanup, the DNA backbone was stained by mixing the labeled DNA with DNA Stain solution (Bionano Genomics) in presence of 1× Flow Buffer (Bionano Genomics) and 1× DTT (Bionano Genomics), and incubated overnight at room temperature. The DLS DNA concentration was measured with the Qubit dsDNA HS Assay (Invitrogen).

Labelled and stained DNA was loaded on the Saphyr chip. Loading of the chip and running of the Bionano Genomics Saphyr System were all performed according to the Saphyr System User Guide (30247 - Bionano Genomics). Data processing was performed using the Bionano Genomics Access software (<https://bionanogenomics.com/support-page/bionano-access-software/>). A total of 1.1 Tb of data was generated. From this data, molecules with a size larger than 150 kb, the threshold for map assembly, represent 356 Gb of data. These filtered data (> 150 kb), corresponding to 651x coverage of the 550 Mb estimated size of *A. pisum* genome, were compiled from 1,557,530 molecules with N50 of 223 kb and an average label density of 13.2/100kb. The filtered molecules were aligned using RefAligner with default parameters. It produced 127 genome maps with a N50 of 20 Mb for a total genome map length of 778.5 Mb. As the map size was longer than expected, due to the heterozygosity, we purged it in order to obtain only one haplotype for each optical map before the hybrid scaffolding step. For that, we first used runCharacterise from Bionano tools to align maps with each other, and created an alignment file (xmap file). From that file, we recovered supernumerary maps which align globally to other maps, with an in-house java program. This way, we purged the 778.5 Mb optical maps and obtained a genome map length of 527 Mb (consistent with the pea aphid genome size), consisting in 34 maps.

For PacBio sequencing, high molecular weight DNA was extracted from the CP lineage (LSR1) following a protocol similar to the one used for ONT sequencing of the OP clone (see above). PacBio genomic DNA libraries were prepared using the SMRTbell Template Prep Kit 1.0 (PacificBiosciences) following the manufacturer’s protocol and sequenced with six SMRTCells 1M on PacBio Sequel (PacificBiosciences) in Gentyane Platform (Clermont-Ferrand, France) and Centre for Genomic Research (University of Liverpool, UK). The raw data (see Table 1 below) are publicly available on NCBI (BioProject ID PRJNA745262). PacBio raw reads were first treated to produced CCS with the ccs program of the suite PacificBioSystems Pitchfork v3.0 (commit 96f0b06, https://github.com/PacificBiosciences/pitchfork) with the option --maxLength 20000. The resulting CCS and subreads from reads with no CCS were mixed. ONT sequencing was also performed on the LSR1 CP lineage, using the same protocol as described above for the OP lineage.

ONT reads and PacBio subreads were then aligned to the *Buchnera aphidicola* strain APS complete genome (NC_002528.1) with minimap2 v2.17 (Li 2018) with default parameters. The reads matching *B. aphidicola* genome were removed for further analyses. The final set of reads was then assembled and polished with flye v2.7.1 (Kolmogorov, et al. 2019) following the website instructions (https://github.com/fenderglass/Flye) for a mix of ONT and PacBio sequences (assembly of the 2 sets with the options -g 530m - iterations 0, then polishing with PacBio data only with the options -resume from polishing - genome-size 530m).

Then, the 34 reduced optical maps were compared individually to the genome sequences with the hybridScaffold.pl script from Solve3.6.1_11162020 (with the options -B 2 and -N 2), and finally all the sequences resulting of hybrid maps were merged into one genome sequence. Lastly this genome sequence was gapfilled with LR_Gapcloser v1.1 (Xu, et al. 2018) (with the option -s p) and the reads corrected with CANU v1.9 (Koren, et al. 2017).

We refer to this final assembly as the “improved CP assembly” (see suppl. Table 2 below for its main characteristics), which is available at <https://bipaa.genouest.org/sp/acyrthosiphon_pisum/download/genome/LSR1_CP>/.

**Supplementary Table 1.** Sequencing data used for genome assemblies

|  | **PacBio data** | **ONT (Nanopore)** | |
| --- | --- | --- | --- |
| Aphid lineage | LSR1 (cyclically parthenogenetic) | LSR1 (cyclically parthenogenetic) | X6_2 (obligately parthenogenetic) |
| Number of reads | 8,007,204 | 372,636 | 1,330,350 |
| Sequenced bases | 51,786,985,914 | 4,240,655,565 | 15,864,369,067 |
| Genome coverage | 97.7X | 8X | 30X |
| Mean read length (bp) | 6,468 | 11,380 | 11,924 |
| Median read length (bp) | 6,163 | 9,010 | 10,262 |
| N50 (bp) | 8,750 | 17,775 | 16,988 |

**Supplementary Table 2.** Genome assembly statistics. Busco analyses were realized with BUSCO 4.0.6, using the dataset insecta_odb10 (1367 BUSCOs, https://busco.ezlab.org/list_of_lineages.html).

|  | **Improved CP genome** | **OP genome** |
| --- | --- | --- |
| Aphid lineage | LSR1 (cyclically parthenogenetic) | X6_2 (obligately parthenogenetic) |
| Data used | PacBio, Nanopore, Optical map | Nanopore |
| Number of scaffolds | 32 | 3889 |
| Assembly size (Mbp) | 527 | 478 |
| N50 (Mbp) | 60 | 0.955 |
| L50 | 4 | 127 |
| BUSCO - single | 1220 | 902 |
| BUSCO - Duplicated | 63 | 8 |
| BUSCO - fragmented | 13 | 186 |
| BUSCO - Missing | 71 | 271 |

*Identification of the actual chromosomal localization of the 30-kb outlier region found on chromosome 1 on the v3.0 reference genome*

Our genome scan identified two main genomic regions with high F_ST_ values, the main 840-kb X-linked candidate region and a short one (30 kb) on chromosome 1 (located between 73,118,603 and 73,151,851, see Supplementary File 3). Given the abrupt changes in F_ST_ at the border of this 30-kb region, we suspected a genome assembly error. In a previous study, the entire *A. pisum* genome was assigned to the X or autosomes based on ratios of sequencing depth in males (X0) to females (XX) (Jaquiéry, et al. 2018). Using these data, we discovered that the 30-kb region was indeed misplaced on the v3.0 reference genome (Li, et al. 2019) and actually belongs to the X chromosome. We located the 30-kb chr1 region on Super-Scaffold_100003 of the improved CP genome, between positions 20,170,923 and 20,205,127 (see Supplementary File 5A). Super-Scaffold_100003 corresponds to a region on the X chromosome of the v3.0 reference genome (Li, et al. 2019) and the 30-kb region would be localized between positions 60,702,513 and 60,707,859. This places the short region of high F_ST_ at ~2 Mb of the main 840-kb candidate region (which also locates on Super-Scaffold_100003, see Supplementary File 5A). These regions may thus be under the influence of the same locus controlling reproductive mode. However, since the F_ST_ values of this 30-kb region were below the F_ST_ threshold of 0.4, we did not further retain it as a candidate region.

*Structure of the 840-kb candidate region in OP and CP genomes*

Finally, to characterize the genomic structure of the 840-kb candidate region, we located this region in the OP genome and in the improved CP genome. In the improved CP genome, we located the 840-kb candidate region on the Super-Scaffold_100003, between positions 22,415,764 and 23,247,663 (Supplementary File 5A). In the OP genome, the whole 840-kb candidate region was also found on a single scaffold (between positions 249,000 and 1,093,000 on scaffold cgt7). Pairwise alignments of the sequence corresponding to the 840-kb region (flanked by 200 kb on each side) in the 3 different genome assemblies (the v3.0 reference genome, the improved CP genome and the OP genome) were assessed using NUCmer v3.07 from package MUMmer v3.22 (Kurtz, et al. 2004). Alignments were filtered using the script delta-filter to keep optimal correspondence with a minimum length of 1000 bp and a minimum alignment identity of 90. We found that the local assembly of the region corresponding to the 840-kb region was concordant between the improved CP assembly and the v3.0 reference assembly and also with the OP genome assembly (Supplementary File 5B and 4C, see also Figure 4 in the manuscript).

**References**

Jaquiéry J, Peccoud J, Ouisse T, Legeai F, Prunier-Leterme N, Gouin A, Nouhaud P, Brisson JA, Bickel R, Purandare S, et al. 2018. Disentangling the causes for faster-X evolution in aphids. **Genome Biology and Evolution** 10:507-520.

Jaquiéry J, Stoeckel S, Larose C, Nouhaud P, Rispe C, Mieuzet L, Bonhomme J, Maheo F, Legeai F, Gauthier JP, et al. 2014. Genetic control of contagious asexuality in the pea aphid. **Plos Genetics** 10.

Kolmogorov M, Yuan J, Lin Y, Pevzner PA. 2019. Assembly of long, error-prone reads using repeat graphs. **Nature Biotechnology** 37:540-546.

Koren S, Walenz BP, Berlin K, Miller JR, Bergman NH, Phillippy AM. 2017. Canu: scalable and accurate long-read assembly via adaptive k-mer weighting and repeat separation. **Genome Research** 27:722-736.

Kurtz S, Phillippy A, Delcher AL, Smoot M, Shumway M, Antonescu C, Salzberg SL. 2004. Versatile and open software for comparing large genomes. **Genome Biology** 5:R12.

Li H. 2018. Minimap2: pairwise alignment for nucleotide sequences. **Bioinformatics** 34:3094-3100.

Li YY, Park H, Smith TE, Moran NA. 2019. Gene family evolution in the pea aphid based on chromosome-level genome assembly. **Molecular Biology and Evolution** 36:2143-2156.

Ruan J, Li H. 2020. Fast and accurate long-read assembly with wtdbg2. **Nature Methods** 17:155-158.

Wick RR, Judd LM, Gorrie CL, Holt KE. 2017. Unicycler: Resolving bacterial genome assemblies from short and long sequencing reads. **PLoS Computational Biology** 13:e1005595.

Xu G-C, Xu T-J, Zhu R, Zhang Y, Li S-Q, Wang H-W, Li J-T. 2018. LR_Gapcloser: a tiling path-based gap closer that uses long reads to complete genome assembly. **GigaScience** 8.

**Supplementary File 5.** A) Location of the 30-kb chromosome 1 region and of the 840-kb candidate region on Super-Scaffold_100003 from the improved CP genome assembly. B and C) MUMmer alignment plots for the nucleotide sequences from the different genome assemblies corresponding to the 840-kb candidate region (plus 200 kb on each side). The x-axis represents coordinates of the v3.0 reference genome assembly. The y-axis represents coordinates of Super-Scaffold_100003 from the improved CP genome assembly (B) or of scaffold ctg7 from the OP genome assembly (C). The purple vertical dashed lines delimit the 840-kb candidate region.

**Supplementary File 6.** Distribution of *F_ST_* (A) and number of SNPs (B) per 20-kb window sliding by 5-kb steps, and their relationships (C). In panels C and D, only windows with at least 50 SNPs and with *F_ST_* above a given threshold were coloured (green for *F_ST_* >0.2, yellow for *F_ST_* > 0.3 and red for *F_ST_* >0.4). The secondary peak just to the left of the main 840-kb candidate region (comprising four grey dots in panel D) is composed of windows with a low number of SNPs (see panel C, bottom right), which were also not supported by the BayPass *C2* statistic.


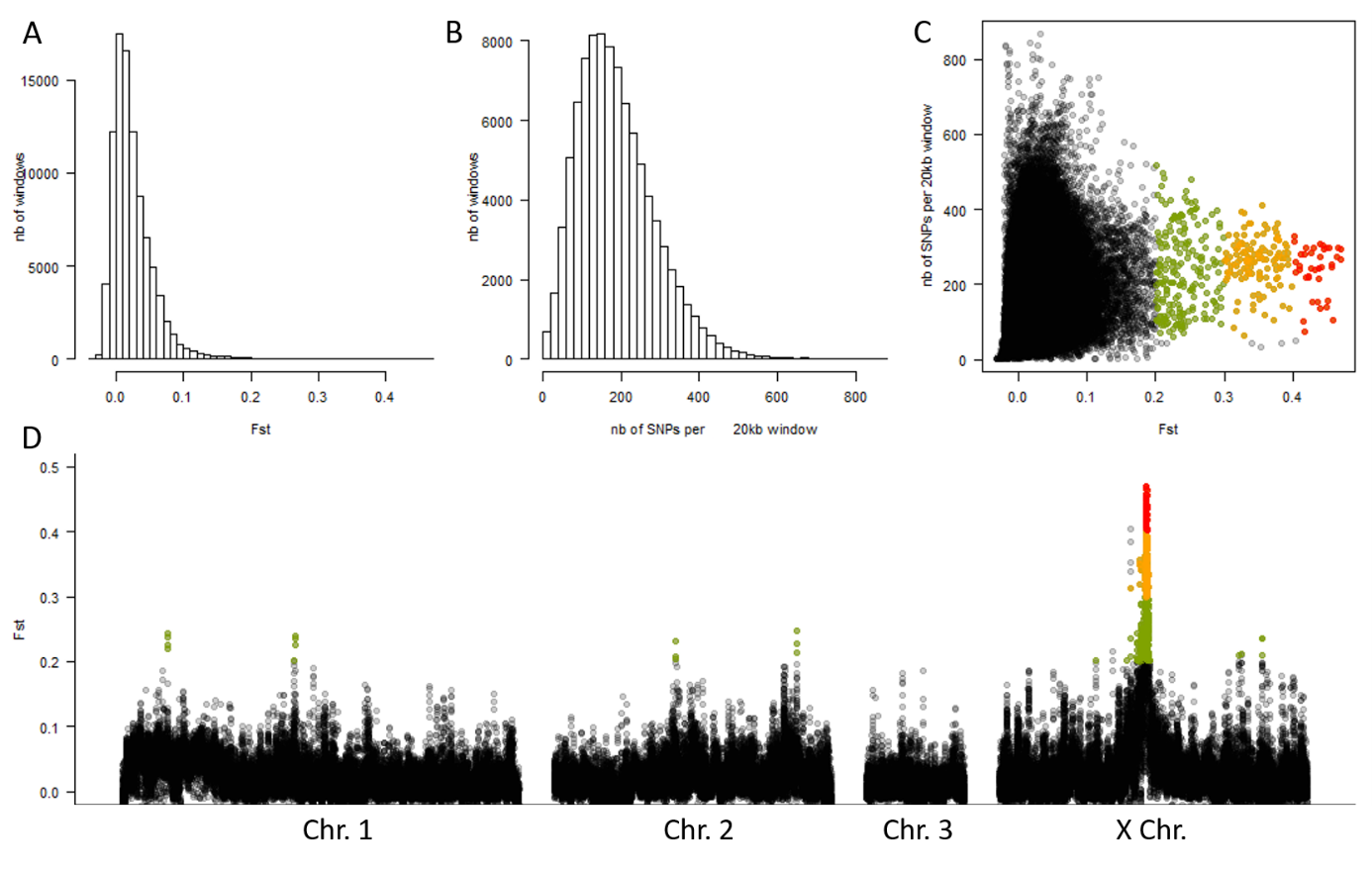


**Supplementary File 7.** Population genetic indices calculated along a region of the X chromosome of *Acyrthosiphon pisum* in non-overlapping 100 kb windows. This region, ranging from 61 to 65.5 Mb, encompasses the main candidate region associated with reproductive mode variation (highlighted in blue) and a locus called *aphicarus* associated with wing polymorphism in males in violet (Braendle, et al. 2005; Li, et al. 2020). A) *F_ST_* between OP and CP populations; B and C) heterozygosity in OP and CP populations, respectively; D and E ) Tajima’s D for OP and CP populations, respectively; F and G) Fay & Wu’s H for OP and CP populations, respectively. Each point (a 100 kb window) was colored according to its *F_ST_* value (red for windows with an average *F_ST_* above 0.3, yellow for *F_ST_* between 0.2 and 0.3, blue for those with *F_ST_* between 0.1 and 0.2, grey for those below 0.1 to facilitate the visualization of the possible relationship between regions with high *F_ST_* values and their values at other indices. *Aphicarus* is characterized by low differentiation (*F_ST_*) between OP and CP populations, and similar patterns for both population types at other indices, namely reduced heterozygosity and Tajima’s D and increased Fay and Wu’s H. On the other hand, particular signatures were only detected in the OP populations for the candidate region associated with the variation in reproductive mode.

**References**

Braendle C, Caillaud MC, Stern DL. 2005. Genetic mapping of aphicarus – a sex-linked locus controlling a wing polymorphism in the pea aphid (*Acyrthosiphon pisum*). Heredity 94:435-442.

Li B, Bickel RD, Parker BJ, Saleh Ziabari O, Liu F, Vellichirammal NN, Simon J-C, Stern DL, Brisson JA. 2020. A large genomic insertion containing a duplicated follistatin gene is linked to the pea aphid male wing dimorphism. Elife 9:e50608.


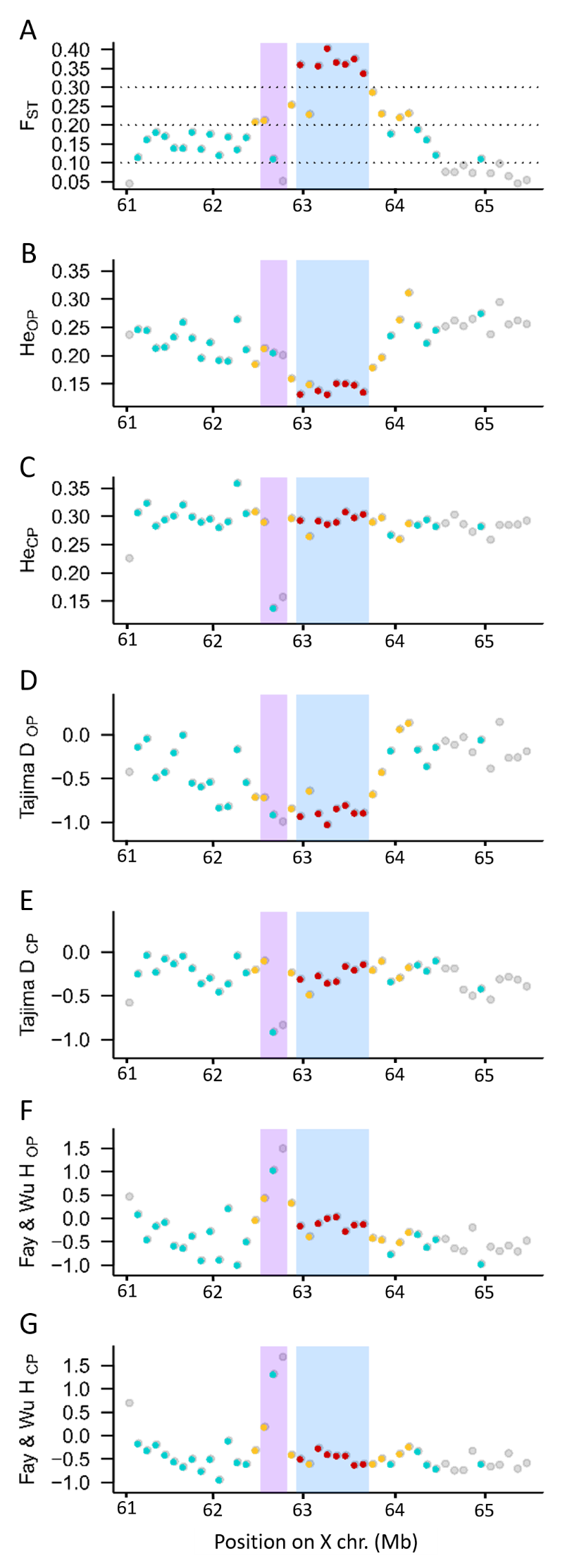


**Supplementary File 8.** Description and annotation of the 32 genes and of the variants with moderate to high impact in the 840-kb candidate region.

*See attached Excel file SupplementaryFile8.xlsx.*

**Supplementary File 9.** Summary of the coverage of the 32 genes located in the candidate region in OP and CP populations.

*See attached Excel file SupplementaryFile9.xlsx.*

**Supplementary File 10.** Detection of genomic regions associated with reproductive mode variation in *Acyrthosiphon pisum* based on the genetic differentiation (*F_ST_*) between obligate parthenogenetic (OP) and cyclical parthenogenetic (CP) populations (100-kb non-overlapping windows). Here, we restricted our analysis to the 181,204 tri-allelic SNPs for which the third allele was supported by only one read. For *F_ST_* calculation, this third allele was ignored and the SNPs were considered biallelic. The 840-kb region identified as candidate using the full dataset is shown in blue. This subset of data composed of tri-allelic SNPs therefore gave results perfectly similar to those obtained with the whole dataset, showing that the inclusion of these 181,204 SNPs does not affect our conclusions.

**
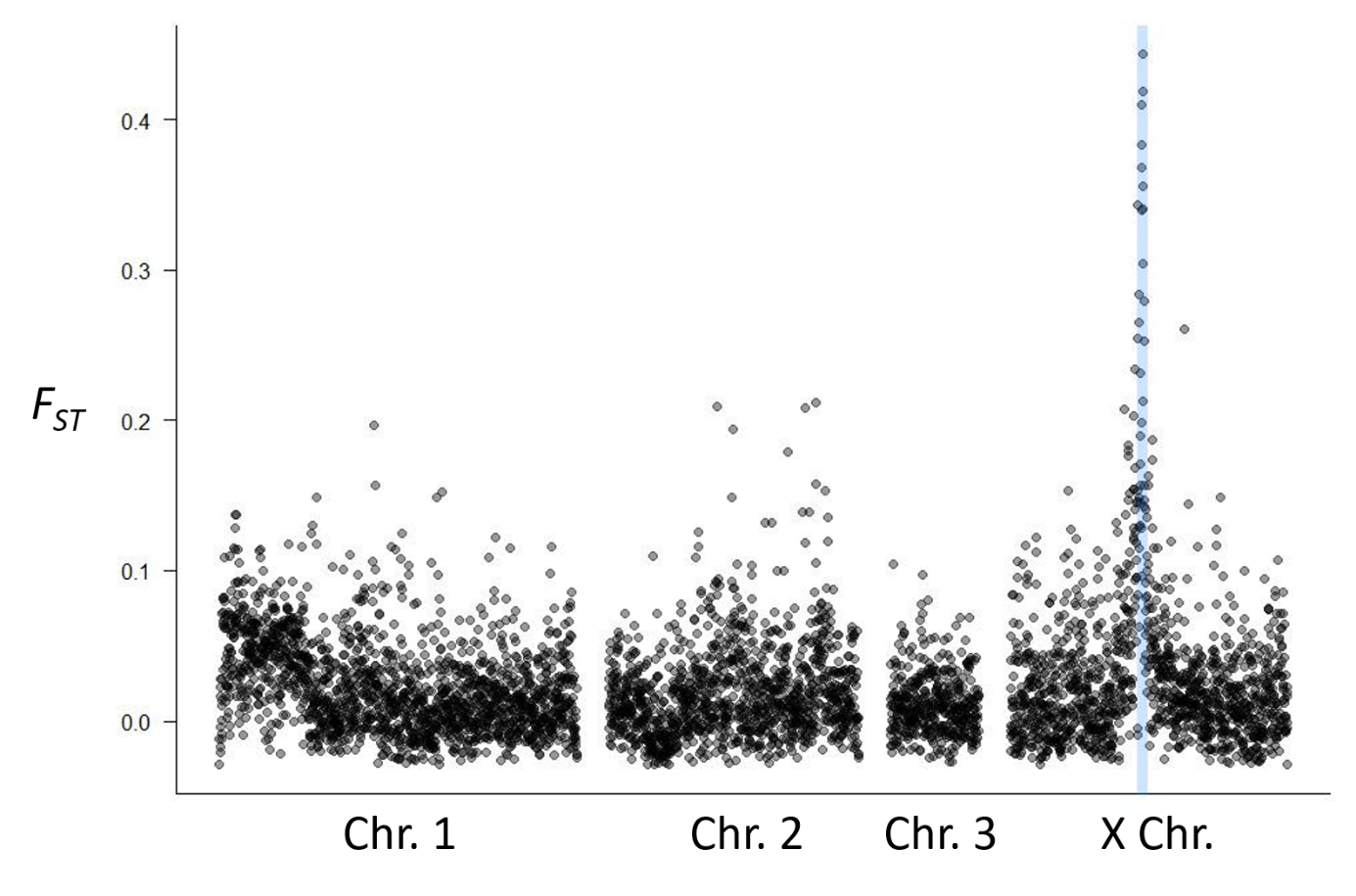
**
